# Adaptive immune changes associate with clinical progression of Alzheimer’s disease

**DOI:** 10.1186/s13024-024-00726-8

**Published:** 2024-04-24

**Authors:** Lynn van Olst, Alwin Kamermans, Sem Halters, Susanne M. A. van der Pol, Ernesto Rodriguez, Inge M. W. Verberk, Sanne G. S. Verberk, Danielle W. R. Wessels, Carla Rodriguez-Mogeda, Jan Verhoeff, Dorine Wouters, Jan Van den Bossche, Juan J. Garcia-Vallejo, Afina W. Lemstra, Maarten E. Witte, Wiesje M. van der Flier, Charlotte E. Teunissen, Helga E. de Vries

**Affiliations:** 1 Department of Molecular Cell Biology and Immunology, Amsterdam UMC Location Vrije Universiteit Amsterdam, De Boelelaan 1117, Amsterdam, the Netherlands; 2 Amsterdam Neuroscience, Neuroinfection & -Inflammation, Amsterdam, the Netherlands; 3 Amsterdam Institute for Infection and Immunity, Cancer Immunology, Amsterdam, the Netherlands; 4 Cancer Center Amsterdam, Cancer Biology and Immunology, Amsterdam, the Netherlands; 5 Department of Laboratory Medicine, Neurochemistry Laboratory, Amsterdam UMC, Vrije Universiteit Amsterdam, De Boelelaan 1117, Amsterdam, the Netherlands; 6 Amsterdam Neuroscience, Neurodegeneration, Amsterdam, the Netherlands; 7 Amsterdam Cardiovascular Sciences, Atherosclerosis & Ischemic Syndromes, Amsterdam, the Netherlands; 8 Amsterdam Gastroenterology Endocrinology Metabolism, Amsterdam, the Netherlands; 9 Cancer Center Amsterdam, Imaging and Biomarkers, Amsterdam, the Netherlands; 10 Department of Neurology, Amsterdam UMC Location VUmc, Vrije Universiteit Amsterdam, Amsterdam, the Netherlands; 11 Amsterdam Institute for Infection and Immunity, Inflammatory Diseases, Amsterdam, the Netherlands; 12 Department of Epidemiology & Data Science, Amsterdam UMC Location VUmc, Vrije Universiteit Amsterdam, Amsterdam, the Netherlands; 13 Amsterdam Cardiovascular Sciences, Microcirculation, Amsterdam, the Netherlands; 14 Amsterdam Neuroscience, Neurovascular Disorders, Amsterdam, the Netherlands; 15 Present address: The Ken & Ruth Davee Department of Neurology, Northwestern University Feinberg School of Medicine, Chicago, IL USA

**Keywords:** Alzheimer’s disease, T cells, TEMRA cells, Adaptive immunity, APOE, Neuroinflammation

## Abstract

**Background:**

Alzheimer’s disease (AD) is the most frequent cause of dementia. Recent evidence suggests the involvement of peripheral immune cells in the disease, but the underlying mechanisms remain unclear.

**Methods:**

We comprehensively mapped peripheral immune changes in AD patients with mild cognitive impairment (MCI) or dementia compared to controls, using cytometry by time-of-flight (CyTOF).

**Results:**

We found an adaptive immune signature in AD, and specifically highlight the accumulation of PD1^+^ CD57^+^ CD8^+^ T effector memory cells re-expressing CD45RA in the MCI stage of AD. In addition, several innate and adaptive immune cell subsets correlated to cerebrospinal fluid (CSF) biomarkers of AD neuropathology and measures for cognitive decline. Intriguingly, subsets of memory T and B cells were negatively associated with CSF biomarkers for tau pathology, neurodegeneration and neuroinflammation in AD patients. Lastly, we established the influence of the APOE ε4 allele on peripheral immunity.

**Conclusions:**

Our findings illustrate significant peripheral immune alterations associated with both early and late clinical stages of AD, emphasizing the necessity for further investigation into how these changes influence underlying brain pathology.

**Supplementary Information:**

The online version contains supplementary material available at 10.1186/s13024-024-00726-8.

## Highlights

• Peripheral CD8^+^ TEMRA cells expressing markers associated with senescence accumulate in AD patients before dementia onset.

• Peripheral immune cells correlate with AD biomarkers, varying by clinical AD stage.

• APOE ε4 modifies peripheral immunity and its association with clinical AD measures.

**Supplementary Information:**

The online version contains supplementary material available at 10.1186/s13024-024-00726-8.

## Background

Alzheimer’s disease (AD) is a neurodegenerative disorder characterized by tau tangles and amyloid-beta (Aβ) deposits in the brain, ultimately leading to neurodegeneration and cognitive decline. The most important genetic risk factor for AD is carrying the apolipoprotein (APOE) ε4 allele, which is associated with increased Aβ accumulation [1–3] and decreased vascular function [4, 5]. While the important role of microglia in AD is nowadays widely accepted [6], accumulating evidence also associates altered levels of adaptive immune cells with dementia [7] and AD [8], and with APOE ε4 status [9]. Research in the last decade has elucidated the existence of meningeal lymphatic vessels and a lymphatic drainage system for CNS antigens, regulating the CNS adaptive immune response [10, 11]. Disruption of these meningeal lymphatics aggravated meningeal Aβ deposition and neuroinflammation in an AD mouse model, signifying the importance of brain lymphatic innervation and an adaptive immune response for AD. Moreover, multiple studies have so far identified infiltrated adaptive immune cells in the brains of AD patients, and the spatial association of these immune cells with neurons, microglia, and Aβ and tau pathology [12, 8, 13–19]. Specifically, CD8^+^ T cells were increased in both the cerebrospinal fluid (CSF) and the hippocampus of AD patients, often located adjacent to Aβ plaques [8, 18] or in the perivascular space of blood vessels with Aβ deposits [8]. In addition, higher numbers of circulating effector memory CD8^+^ T cells re-expressing CD45RA were observed in AD patients and were associated with worse cognition [8]. Together, these studies provide strong evidence that AD is accompanied by changes in adaptive immune cells in both the circulation and the brain.

In the last years, research has moved increasingly toward the pre-dementia phase of AD, particularly to the mild cognitive impairment (MCI) stage, during which there is a measurable loss of cognitive ability and some brain atrophy, but the patient is still capable of independent living. Immune profiling at this stage has not been carried out in great detail but is of high importance if one wants to understand possible immunological associations with early cognitive decline. As such, the present study aimed to characterize the circulating immune cell subsets in both MCI and dementia stages of AD and to explore immunological associations with clinical parameters such as CSF analyses of Aβ and tau pathology, CSF markers for neurodegeneration and neuroinflammation, and different cognitive tests covering global cognition, memory, attention, and executive function. In addition, we have included the potential effects of the APOE ε4 genotype on the established immune signature of AD patients. Altogether, we investigated the peripheral immune landscape of AD patients in the early and late stages of the disease and found adaptive immune changes related to cognitive and biological pathology.

## Methods

### Study population

We included 115 subjects from the Amsterdam Dementia Cohort [20] with a baseline diagnosis of subjective cognitive complaints (e.g. controls, *n* = 35), MCI-AD (*n* = 21), or AD dementia (*n* = 59) (Additional file 1). Subjects visited the Alzheimer's Center in Amsterdam between May 2018 and March 2020 for standardized dementia screening consisting of neurological, physical, and neuropsychological evaluation and brain magnetic resonance imaging (MRI). In addition, a lumbar puncture was offered. Diagnoses of MCI and dementia were made according to the NIA-AA criteria in a multidisciplinary consensus meeting according to the then-applicable guidelines. The label of subjective cognitive decline was assigned when no abnormalities were observed on clinical or cognitive tests and when the criteria for MCI, dementia, or other medical conditions and psychiatric disorders that could potentially cause cognitive deficits were not met. Participants were only included if CSF measurements of Aβ42, tau phosphorylated at threonine 181 (pTau), and total tau (tTau) were available. Control patients were required to have a Mini-Mental State Examination (MMSE) score of ≥ 24, except for one control who had an MMSE of 20 because the subject was illiterate. Cognitive tests were not used for this individual. In addition, MCI-AD and AD dementia patients were selected to have positive CSF levels of Aβ and ρTau. Individuals with subjective cognitive decline with negative CSF levels of Aβ, and ρTau served as controls. The research was approved by the medical ethical committee of the VU Medical Center and was following the Helsinki Declaration of 1975. All subjects provided written informed consent to use medical data and biomaterials for scientific research.

### Cognitive assessment

We used the following test to measure cognitive function: global cognition was assessed by the MMSE and the Montreal Cognitive Assessment (MOCA). Memory was assessed by the Dutch version of the Rey Auditory Verbal Learning Test (RAVLT) with immediate recall, delayed recall, and recognition. Trail Making Test (TMT) A and B provided a measure of attention and executive function.

### CSF biomarker measurements

CSF concentrations of amyloid-beta 1–42 (Aβ42), pTau and tTau were measured with Elecsys or using Innotest ELISAs (Fuijirebio, Ghent, Belgium) by trained technicians who were blinded for clinical diagnosis. Levels were subsequently dichotomized for Aβ (abnormal Aβ42 ≤ 1000 pg/ml Elecsys or ≤ 813 pg/ml Innotest), pTau (abnormal ρTau ≥ 19 pg/ml Elecsys or ≥ 52 pg/ml Innotest) and tTau (abnormal tTau ≥ 235 pg/ml Elecsys or ≥ 375 pg/ml Innotest). Correlations with the abundance of immune cell subsets were only analyzed in participants whose immune cells were isolated on the same day as the lumbar puncture for the biomarker measurement was performed (excluding 8 patients) and the measurements were performed by Elecsys (excluding 1 patient). CSF sTREM2 measurements were determined using Innotest solid-phase enzyme immunoassays (Fuijirebio, Ghent, Belgium) and technically validated in-house for the detection of sTREM2 in CSF with optimal assay performance for parallelism, dilution-linearity, and recovery analysis. CSF YKL-40 measurements were done by enzyme-linked immunosorbent assay (ELISA; Quidel, San Diego, CA, USA), as validated before [21]. CSF NfL measurements were done with the NfL advantage kit according to the manufacturer’s instructions, and commercially available from Quanterix, Billerica, Massachusetts. CSF sTREM-2, YKL-40, and NfL were measured in CSF samples from participants whose immune cells were isolated on the same day as the lumbar puncture for the biomarker measurement was performed.

### APOE genotyping

Sequencing was performed in EDTA plasma using Sanger sequencing on ABI130XL, after DNA amplification by PCR technique and analysis for size and quantity by QIAxcel DNA Fast Analysis Kit (Qiagen). APOE ε4 carriers had one or two APOE ε4 copies, whereas ε4 non-carriers had none.

### Immune cell isolations

Blood was drawn before 2:00 PM in Vacutainer Mononuclear Cell Preparation Tubes with Sodium Citrate (CPT, BD Biosciences) and isolated according to the manufacturer’s instructions. Briefly, CPT tubes were centrifuged for 30 min at 1,800 g with brake 9 at RT. Tubes were then gently inverted so the mononuclear cells and platelets were resuspended in the plasma layer. The content of the CPT tube was then poured into a 15 mL tube, and the remaining immune cells in the CPT tube were recovered by rinsing the tube with PBS, which was then added to the 15 mL tube. The 15 mL tube containing the isolated immune cells was filled up with PBS, inverted, and centrifuged for 10 min at 300 g with brake 9 at RT. The supernatant was carefully removed, and the cell pellet was resuspended in another 15 mL of PBS, inverted, and centrifuged for 10 min at 300 g with brake 9 at RT. After the supernatant was carefully removed, the cell pellet was resuspended in 4 mL of PBS of which a sample of 300 µL was taken and analyzed for cell abundance on a Sysmex XN-9000. The 15 mL tube was filled up with PBS, inverted, and centrifuged for 10 min at 300 g with brake 9 at RT. Cell pellets were resuspended at a concentration of 5 × 10^6^ cells per mL in 10% DMSO/FCS and put in a Corning CoolCell alcohol-free freezing container at -80 °C for 24 h. Samples were later transferred and stored in liquid nitrogen until staining and sample acquisition.

### CyTOF antibody labeling and titration

Antibody labeling with the indicated metal tag was performed using the MaxPAR antibody conjugation kit (Fluidigm) according to the manufacturer’s instructions. Purification of the bound antibody was performed with high-performance liquid chromatography (Thermo Fisher) and subsequently concentrated by filtering with a 10-kDa filter (Merck Millipore) in a swing-out bucket at 4000 RPM for 15 min. The end volume was determined and an equal volume of antibody stabilizer buffer (Fluidigm; supplemented with 0.05% sodium azide) was added before the antibodies were stored at 4 °C. All antibodies used in this study were titrated using both fixed and unfixed thawed immune cells and the most optimal concentrations with the least spillover were chosen. We adjusted the concentration accordingly for our frozen antibody cocktail aliquots.

### CyTOF antibody-cocktail aliquots

CyTOF antibody cocktails were prepared as described before [22]. Briefly, conjugated antibodies were combined in a surface and nuclear antibody cocktail in the appropriate ratios (Additional file 2) without further dilution in cell staining buffer (CSB) (Fluidigm, #201,068). The surface and nuclear antibody cocktails were centrifuged for 15 min at 15,000 g at 4 °C to exclude antibody aggregates. Then, supernatants were transferred to new tubes and divided over several aliquots (surface cocktail: 50, 75, 150, and 175 µL aliquots; nuclear cocktail: 20, 15, 10, and 5 µL aliquots). Tubes were tightly closed and further sealed with parafilm and directly placed at − 80 °C. Before use, aliquots were thawed at RT. Once liquid, the cocktails were centrifuged for 15 min at 15,000 g at 4 °C. Then, the supernatant was transferred to the cells and resuspended in the appropriate buffer in a cell-to-antibody ratio of 3 × 10^6^ cells per 100 μL in CSB; as described in the CyTOF staining.

### Generation of the CyTOF reference sample

The reference sample contained immune cells obtained from the blood of 3 control donors of which a part was stimulated with a cytokine cocktail to induce expression of each protein and transcription factor included in the CyTOF panel. Unstimulated and stimulated PBMCs were combined, stained for viability as described earlier, fixated, and stored in aliquots at -80 °C until further use.

### CyTOF staining

Reagents were cooled on ice, and centrifugation steps were performed at 1500 RPM before cell fixation and 800 g after fixation, for 7 min at 4 °C (9 acc/7 dec). Incubations were performed at RT. Samples (approximately 1–3 million cells per sample) were thawed rapidly and were transferred to a 15 mL tube with 1 mL of chilled Fetal Calf Serum (FCS, Corning, #35–079-CV). Then, 10 mL of RP10 (RPMI 1640, 10% FCS, 1% penicillin/streptomycin, 1% glutamine) was added to each tube, samples were centrifuged, and the supernatant was removed. Samples were then resuspended in 100 µL of MaxPar PBS (Fluidigm, #201058) and transferred to a 96-V-bottom plate. The samples were centrifuged and washed with 150 μL of MaxPar PBS. Samples were stained with the viability marker Cell-ID™ Cisplatin-198Pt (1:1000, Fluidigm, #201198) in MaxPar PBS for 7.5 min at 37 °C. Then, 25 µL of FCS was added to each sample, cells were centrifuged, and the supernatant was removed. Cells were then washed with 150 µL of MaxPar PBS and fixated with 1 mL of freshly made 1.6% PFA (Thermo Fisher, #28906) in Maxpar PBS for 10 min. Reference samples (2 per barcode) were added to the 96-V-bottom plate, and cells were washed twice with 150 μL of 1X Barcode Perm Buffer (Fluidigm, #201057) and incubated with the appropriate palladium barcodes (Fluidigm, #201060) in 1X Barcode Perm Buffer for 30 min. After centrifugation, samples were washed twice with 150 μL of CSB and the cells from all samples were pooled. Then, an aliquot of the surface antibody cocktail (Additional file 2) was thawed and centrifuged for 15 min at 15,000 g at 4 °C. At the same time, cells in the combined sample were counted and after centrifugation, incubated with Human TruStain FcX Fc receptor blocking solution (BioLegend, #422302) diluted in CSB (1:50) for 10 min. The supernatant of the centrifuged antibody cocktail was then added to the combined sample in a cell-to-antibody ratio of 3 × 10^6^ cells per 100 uL in CSB. This was followed by a 30 min incubation. After the incubation cells were washed twice with CSB, followed by a wash with Maxpar PBS. Then, cells were fixated with 1 mL of freshly made 1.6% PFA (Thermo Fisher, #28906) in Maxpar PBS for 10 min. After centrifugation, cells were permeabilized with 1 mL of FoxP3 Fix/Perm working solution (eBioscience, #00–5523) for 30 min, followed by two washes with 1X Permeabilization Buffer (eBioscience, #00–5523). Then, a thawed and centrifuged nuclear antibody cocktail (Additional file 2) in 1X Permeabilization Buffer was added in a cell-to-antibody ratio of 3 × 10^6^ cells per 100 μL and incubated for 45 min. Cells were then washed three times with 1X Permeabilization Buffer and fixated with 1 mL of freshly made 1.6% PFA in Maxpar PBS for 10 min. After centrifugation, nucleated cells were stained with Maxpar Intercalator (Fluidigm, #201192B) diluted 1:4000 in Maxpar Fix and Perm Buffer (Fluidigm, #201067) and incubated overnight at 4 °C, until sample acquisition.

### CyTOF sample acquisition

Cells in Maxpar Intercalator solution were washed twice with CSB and divided over approximately 1 × 10^6^ cells per tube, followed by two washes with cell acquisition solution (CAS) (Fluidigm, #201240) right before acquisition. Samples were filtered and calibration beads (Fluidigm, #201078) were added to the suspension to 15% of the final volume. Cells were acquired on a Helios™ (Fluidigm), with an event rate of 250 – 350 events per second in CAS. Runs took approximately 30 – 45 min. During the day, tuning of the machine was performed during start-up and after 4 h of sample acquisition. Within each barcoded set of samples, two reference samples were included to correct for differences in staining intensity between barcodes due to technical variation in the staining protocol or daily changes in instrument functioning, as will be discussed later.

### CyTOF data analysis

Acquired samples were randomized using Gaussian negative half zero randomization, normalized using bead normalization, and concatenated using the CyTOF Software version 6.7. Barcoded FCS files were then uploaded into OMIQ data analysis software (https://www.omiq.ai/) and normalization beads, cell debris, and cell doublets were removed from the data per barcode using the bead intensity, DNA staining, and Gaussian parameters. In addition, cells with positive reactivity for CD45-89Y were selected. There was an upward drift in the background levels of CD3-141Pr, and segments with the highest background increase were manually removed per barcode. Then we exported new ‘cleaned’ FCS files and used the flowCut algorithm (https://github.com/jmeskas/flowCut) which automatically removes outlier events in flow cytometry data files due to abnormal flow behaviors resulting from clogs and other common technical problems and included every channel except CD3-141Pr, CD4-145Nd, and CD8a-146Nd. After flowCut, all channels were inspected carefully and another pre-gating round wherein we selected stable flow using CD28-158Gd was performed manually in OMIQ per barcode. Then, samples were debarcoded using the CyTOF Software version 6.7. Per debarcoded FCS file, live immune cells were selected using negative reactivity for viability marker Cell-ID Cisplatin-195Pt and positive reactivity for CD45-89Y, and batch alignment was performed on the pre-gated, live CD45^+^ debarcoded FCS files using CytoNorm [23]. For CytoNorm, 50,000 cells were imputed per sample and xdim = 10, ydim = 10, and nClusters = 10 were used as settings. The pre-gating strategy can be viewed in Fig. S1. The processed FCS files were then uploaded into OMIQ data analysis software and data analysis was performed. Here, T cells were pre-selected by positive reactivity for CD3-141Pr and negative reactivity for CD19-165Ho. From the negative gate (e.g. non-T-cells), B cells were pre-selected using positive reactivity for CD19-165Ho and negative reactivity for CD11b-156Gd. The ‘left-over’ cells that had negative reactivity for CD3-141Pr and CD19-165Ho were imputed as a ‘rest’ population and were later known to contain DCs, monocytes, and NK cells. Of the pre-selected T cells, B cells, and ‘rest’ cells a maximum of 20,000 cells per sample were imputed, which led to a total of 3,204,588 T cells, 2,288,834 B cells, and 3,168,876 DCs, monocytes, NK cells that were used for the downstream analysis. Data were visualized in UMAPs and tSNEs and cells were appointed to clusters by Phenotyping by Accelerated Refined Community (PARC) [24]. Visual inspection of 1) PARC-derived clusters overlaid on corresponding UMAPs and tSNEs derived from the same sample, and 2) clustered heatmaps that compare median marker expression between clusters, guided the manual merging of clusters and their annotation into biologically relevant immune cell subsets. In addition, we did not further analyze the abundance of clusters of unknown origin, or of non-T-cell, non-B-cell, and non-DC/monocyte/NK-cell clusters that surpassed the pre-gating strategy and were found within the pools of pre-selected T cells, B cells and ‘rest’ cells respectively. Importantly, these clusters of cells are still part of the ‘total’ amount of immune cells of which the fraction of each annotated cluster is calculated.

### Immunohistochemistry

Sections of the middle temporal gyrus were defrosted at RT and subjected to epitope retrieval using citrate buffer pH 6.0 at 95 °C for 30 min in a water bath. Slides were blocked using 1% bovine serum albumin (BSA) in PBS with 0.05% Tween-20 for 30 min. Sections were then incubated with a primary antibody (Additional file 15) in 10 × diluted blocking buffer for 60 min at RT followed by a 60 min incubation with Alexa fluorophore-labeled secondary antibodies (Thermo Fisher Scientific). Sections were counterstained for DNA using DAPI (1:10,000, Molecular Probes). After washing, sections were embedded in Mowiol mounting medium and stored in the dark at 4 °C until image acquisition. Slides were imaged using the Vectra 3.0 spectral imaging system (PerkinElmer), with low magnification to get an overview of the slide and to measure the total tissue area. Next, tissues were manually scanned in a systematic fashion using a Leica TCS SP8 HyD confocal microscope. Each encountered CD8^+^ cell that was localized outside of vessels (as determined by ULEX staining) was counted and imaged with a 0.037 µm/pixel resolution to determine if the cell expressed CD57. Cell counts were then normalized to the total tissue area. Staining, imaging, and analysis were performed blinded. All parties received permission to perform autopsies, for the use of tissue and access to medical records for research purposes, following the corresponding ethical statements (project nr. 1423). The informed consent forms of the Netherlands Brain Bank have been approved by the Medical Ethics Committee of the VU Medical Center in Amsterdam (2009/148). Relevant clinical information of the donors is summarized in Additional file 8.

### Plasma cytokine and chemokine measurements

Nonfasted EDTA plasma samples were procured via venipuncture and centrifuged within an average time frame of 2 h post-collection. This centrifugation was performed at 1,800 g for 10 min at RT. Subsequently, the samples were stored at -80 °C pending further analysis. We determined the levels of various cytokines and chemokines in blood plasma using the bead-based Human CD8/NK panel V02 (including IL17A, IL2, IL4, IL10, IL6, TNFα, IFNγ, Granzyme A, Granzyme B, Perforin) and the Human custom panel (comprising TNFα, IL5, IL13, CXCL10, IFNα, CCL2, CXCL8, CXCL9, CXCL13, CXCL12, IFNβ, TGFβ) from the LEGENDplex kit (BioLegend). This involved diluting plasma two-fold and incubating it overnight at 4 °C with beads specific to these cytokines and chemokines on a plate shaker. After incubation, biotinylated detection antibodies were added to the beads for one hour at RT on a plate shaker. Next, the samples were incubated with PE-conjugated streptavidin for 30 min at RT on a plate shaker. Following a final wash, the samples were analyzed using the AttuneTM CytPix™ Flow Cytometer. A standard curve was created using serial dilutions of standard controls provided by the manufacturer. The LEGENDplex™ Data Analysis Software Suite from Qogni was used to calculate the specific cytokine and chemokine levels. For cytokines with concentrations beyond the detection limit, we used the software's "Show Concentrations outside LOD" function to estimate their levels. Cytokines where over 50 percent of samples were below the detection limit were excluded from further analysis. Details on sample selection for LEGENDplex analysis are provided in Additional file 13.

### Gene analysis using the nCounter metabolic pathways panel

RNA purity was investigated using eukaryotic RNA analysis on RNA ScreenTape (Agilent Technologies). The data were analyzed with TapeStation Analysis Software 4.1 and RNA Integrity Number (RINe) was approved when above 6.5 on a scale from 1 to 10. Approximately 1,000,000 lysed PBMCs were hybridized to a nCounter Metabolic Pathways panel for profiling 768 genes human genes across 34 annotated pathways at 65 °C overnight (NanoString Technologies). Hybridized samples were processed on a nCounter prep station and data were collected on a nCounter digital analyzer (NanoString Technologies), following the manufacturer’s instructions. Raw data was imported into nSolver 4.0 (NanoString Technologies) for data quality checks, background thresholding, and normalization. The background level was determined by the geometric mean counts of the eight negative control probes. Positive control normalization was determined by the geometric mean counts of the six positive control probes. Samples that contained fewer than 50% of probes above background or that had imaging or positive control linearity flags were excluded from further analysis. Probes that have raw counts below background in all samples were excluded from differential expression analysis to avoid false-positive results. Data were normalized by the geometric mean of housekeeping genes. Samples selected for NanoString analysis are outlined in Additional file 14.

### Statistics

Counts per immune cell subset were exported from OMIQ and their fractions of the total amount of immune cells per individual were calculated. For the multivariate general linear model (GLM), fractions of immune cells were logit transformed LN ( *fraction* / ( 1 – *fraction*)), and 0.01 was added before transformation to each fraction of CD45RA^+^ CD57^+^ γδ T cells as one individual was devoid of cells in this cluster. Logit transformed clusters were imputed as dependent variables, diagnosis (e.g. control, MCI, Dem) as a fixed factor, and age and sex were used as covariates in IBM SPSS Statistics 28. The main effects were compared using the Estimated Marginal Means with Sidak adjustment for the confidence intervals. We found 2 possible multivariate outliers using the Mahalanobis Distance, but since none influenced the results, we did not remove them. Subsets of immune cells are highly correlated, and hypotheses are interdependent technically and biologically (for example, subsets of interrelated immune cells, as the ‘parent’ CD8^+^ T cells and the ‘child’ CD8^+^ TEMRA cells). Therefore, we have provided the raw *P* values (unless stated otherwise) and adjusted the* P*-value using Sidak if more than 2 groups (e.g. control, MCI, Dem) were compared. Untransformed data were used for the Spearman correlation networks in R and IBM SPSS Statistics 28, and the Pattern Hunter analysis at https://www.metaboanalyst.ca/. Spearman correlation networks were identified per experimental group, and the *P*-value was adjusted using a false discovery rate of 5% in R. Partial two-tailed Spearman correlations between immune cell subsets and disease outcome measures were identified separately in the experimental groups as stated in the text and controlled for age and sex in IBM SPSS Statistics 28. Missing values were excluded pairwise. Correlations with *P* < 0.01 were considered significant. For the immunohistochemical analyses, GraphPad Prism 8.2.1 was used and the Shapiro–Wilk, and F tests were applied to test for normality and equality of variances, respectively, and appropriate tests were selected accordingly. For the number of CD8^+^ T cells per area between control and AD groups, an unpaired two-tailed Student’s t-test with Welch’s correction for unequal variances was used, and the Mann–Whitney test was used for comparing the number of CD8^+^ CD57^+^ T cells per area between control and AD groups. Data were judged to be statistically significant when *P* < 0.05. For NanoString analysis, comparisons per gene between the different APOE genotypes, we used a multivariate general linear model (GLM) with sex and age as covariates in IBM SPSS Statistics 28, the *P*-value was adjusted using a false discovery rate of 5%. Log(fold change) and -log10(*P*-value) were plotted using GraphPad Prism 8.2.1 to create a volcano plot.

## Results

### Circulating CD57^+^ CD8^+^ TEMRA cells increase in the MCI stage of AD

**Fig. 1:**
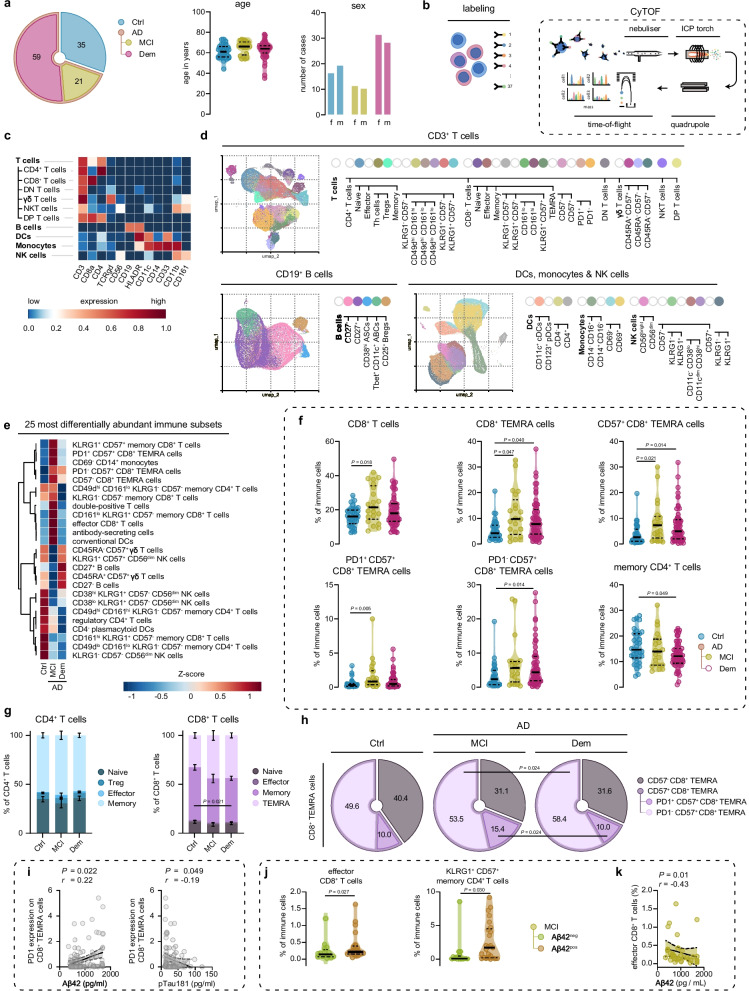
Peripheral CD8^+^ TEMRA cells accumulate in AD patients before the onset of dementia. **a** Cohort demographics. **b** Experimental overview **c**. Heatmap showing median expression values of selected markers for the major immune cell populations. **d** UMAPs displaying pre-gated T cells, B cells and DCs, monocytes and NK cells from the blood of control and AD cases. Colors correspond to PARC-guided clustering. **e** Heatmap showing the top 25 populations with differential abundance between groups measured by ANOVA. **f** Violin plots displaying the percentage of significant different immune cell subsets out of the total CD45^+^ immune population using a GLM with age and sex as covariates. **g** Stacked bar graphs showing the percentage of CD4^+^ and CD8^+^ T cell subpopulations of total CD4^+^ T cells and CD8^+^ T cells using a GLM with age and sex as covariates. **h** Pie charts showing the percentage of CD8^+^ TEMRA subpopulations of total CD8^+^ TEMRA cells. **i** Correlation graph showing the association between PD1 expression on CD8^+^ TEMRA cells and CSF-derived Aβ42 (left) and pTau181 (right) levels using a Spearman's rho correlation with age/sex correction in the entire cohort. **j** Violin plots displaying the percentage of significant different T cell subsets out of the total CD45^+^ immune population using a GLM with age and sex as covariates between Aβ-negative and –positive patients with MCI. **k** Correlation graph showing the association between effector CD8^+^ T cells and CSF-derived Aβ42 levels using a Spearman's rho correlation with age/sex correction. **f**, **j** Violin plots show median ± quartiles. **i**, **k** Correlation plot show linear regression ± 95% confidence intervals; **a**-**k**. *n* = 115 in total, *n* = 35 of control, *n* = 21 of MCI due to AD, *n* = 59 of Dem,* n* = 16 Aβ^−^ MCI spectrum, *n* = 25 Aβ^+^ MCI spectrum; 3,204,588 CD3^+^ T cells, 3,168,876 DCs, monocytes & NK cells and 2,288,834 CD19^+^ B cells were imputed in the clustering analyses; GLM = multivariate general linear model; Ctrl = Control, MCI = mild cognitive impairment; Dem = ; AD = Alzheimer’s disease; TEMRA = terminally differentiated effector memory cells re-expressing CD45RA; DCs = dendritic cells; γδ = gamma-delta; NK = natural-killer

In this study, we examined the peripheral immune landscape of 80 Alzheimer's disease (AD) diagnosed patients (Fig. 1a; S1a; and Additional file 1). Among the cohort, 59 patients were diagnosed with dementia and are herein referred to as 'AD dementia'. The remaining 21 patients were diagnosed with MCI and are designated as 'MCI-AD'. The diagnostic demarcations employed adhered strictly to the National Institute on Aging-Alzheimer's Association (NIA-AA) guidelines and were established through a multidisciplinary consensus meeting [25, 26]. Confirmation of AD diagnosis was accomplished through CSF biomarker analysis, including amyloid-β 1–42 (Aβ42), tau protein phosphorylated at threonine 181 (pTau), and total tau (tTau). Our study also incorporated a control group of 35 cognitively healthy individuals, who were age and sex-matched to the patient cohort and displayed no abnormalities in clinical or cognitive evaluations, nor any anomalous CSF biomarkers related to AD. Isolated peripheral blood mononuclear cells (PBMCs) of control and AD patients were stained with a 37-heavy metal isotope-tagged antibody panel for mass cytometry by time-of-flight (CyTOF) containing surface and nuclear markers to define the major immune cell subsets within PBMCs (Additional file 2). Data were pre-cleaned and PBMCs were divided, through manual gating, into 3 subsets that consisted of (1) CD3^+^ CD19^−^ T cells, (2) CD11b^+^ CD3^−^ CD19^−^ dendritic cells (DCs), monocytes, and natural killer (NK) cells, and (3) CD19 CD11b^−^ CD3^−^ B cells (Fig. S1b, c). Cells were then unsupervised appointed to clusters by Phenotyping by Accelerated Refined Community (PARC) [24] (Fig. 1b, c; Fig. S2; Additional file 3). If needed, PARC-derived clusters were further manually merged into immune cell subsets and tentative names were assigned based on the expression of the 37 markers within the panel.

Using ANOVA, we found that the top-25 immune cell subsets that were most different in abundance between the 3 groups consisted mostly of T cell-derived clusters (Fig. 1d). We then used a multivariate general linear model (GLM) with sex and age as covariates to test for differences in the abundance of all annotated cell types between the AD dementia, MCI-AD and cognitively normal control groups (Additional file 4, Fig. S2). We found that the fraction of CD8^+^ T cells out of the isolated PBMCs was higher in patients with MCI-AD, while CD8^+^ T effector memory cells re-expressing CD45RA (TEMRA) cells, a population with potent pro-inflammatory and cytotoxic effector functions [27], were increased in both patients with MCI-AD and with AD dementia (Fig. 1e,f). Further subtyping of CD8^+^ TEMRA cells revealed that specifically CD57, a marker for senescent T cells, expressing CD8^+^ TEMRA cells were increased in MCI-AD and AD dementia groups (Fig. 1e-g). Of this subset, cells that expressed programmed cell death protein 1 (PD1), an inhibitory receptor [28–30] quickly upregulated after T cell receptor (TCR) stimulation [31], were enriched in patients with MCI-AD while PD1^−^ cells were increased in AD dementia (Fig. 1e). We also observed that CD4^+^ memory T cells were diminished in AD dementia (Fig. 1e,f), and a similar reduction of memory cells was found within the CD8^+^ T cell group in the AD dementia cohort (Fig. 1g). Significantly, PD1^+^ CD57^+^ CD8^+^ TEMRA cells increased within the total population of CD8^+^ TEMRA cells in MCI-AD patients compared to those with dementia (Fig. 1h). We next separated the groups by sex and observed the strongest increase in PD1^−^ CD57^+^ CD8^+^ TEMRA cells in female MCI-AD patients, while males had a higher increase of this T cell population in AD dementia (Fig. S3a). PD1^+^ CD57^+^ CD8^+^ TEMRA cells were increased in both female and male MCI-AD patient groups (Fig. S3a). We further explored PD1 expression by CD8^+^ TEMRA cells in relation to CSF levels of Aβ42 and pTau. This analysis revealed that decreased PD1 expression correlated with lower Aβ42 levels in the CSF (indicative of increased brain Aβ pathology, *P* = 0.022; *r* = -0.22, age and sex corrected) and with higher levels of CSF pTau in the entire cohort (*P* = 0.049; *r* = -0.19, age and sex corrected: Fig. 1i). Together, these data highlight the accumulation of PD1^+^ CD57^+^ CD8^+^ TEMRA cells during the prodromal phase of AD, and link lower PD1 expression on these cells to increased CSF biomarkers indicative of AD pathology.

These findings encouraged us to further investigate T cell changes in the early stages of AD. For this analysis, 20 extra MCI patients were included with varying or normal CSF biomarker levels (for characteristics, see Additional file 5), and patients were categorized into two groups based on normal or abnormal CSF-Aβ42 levels. By using a GLM with sex and age as covariates on T cell subsets, we found that CD8^+^ effector T cells and CD57^+^ KLRG1^+^ CD4^+^ terminally differentiated effector memory T cells were higher in MCI patients with amyloid pathology (Fig. 1j). In addition, higher levels of CD8^+^ effector T cells were associated (*P* = 0.01; *r* = -0.43, age and sex corrected) with increased amyloid pathology, as measured by lower levels of CSF-Aβ42, at the total MCI spectrum (Fig. 1k). We did not find a correlation for CSF-Aβ42 or CSF-levels of pTau with other CD8^+^ T cell subsets. The only other (negative) correlation observed was between CD161^hi^ KLRG1^+^ CD57^−^ CD8^+^ effector-like memory T cells and CSF levels of tTau (Fig. S3b). Together, these data show that effector CD8^+^ T cells are associated with AD pathology in subjects diagnosed with MCI.

### CD8^+^ TEMRA cells foster complex immune networks during the MCI stage

**Fig. 2:**
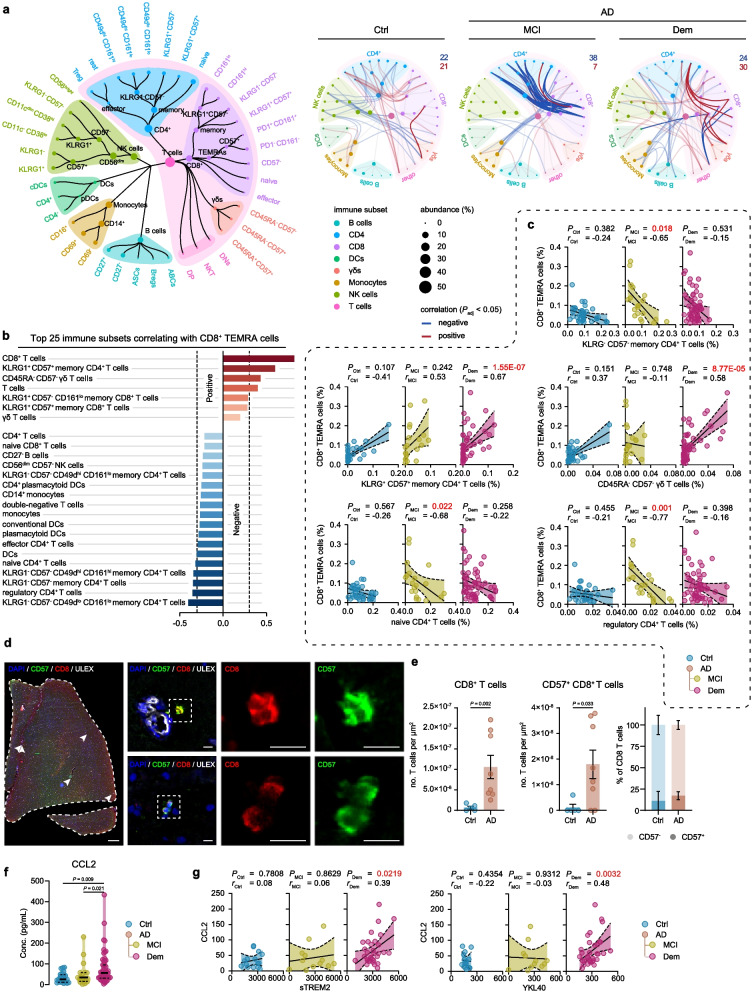
Peripheral CD8^+^ TEMRA cells are inversely correlated to CD4^+^ T cell subsets in AD patients. **a** Spearman correlation network of immune populations with nodes visualizing immune subsets and lines representing correlation coefficients for unique relationships between clusters. The size of the nodes represents the abundance of the population, and the color nodes represent the immune parent. Blue lines and red lines represent respectively negative and positive correlations between connected immune subsets. Darker colors present correlations with CD8^+^ TEMRA subsets. b Graph displays the 25 strongest Spearman correlations between CD8^+^ TEMRA cells and other immune subsets within the cohort. c Correlation graph showing Spearman's rho correlation with age/sex correction of CD8^+^ TEMRA cells with selected immune cell populations. Graph shows linear regression ± 95% confidence intervals. **d** Representative immunohistochemical staining of CD57^+^ CD8^+^ T cells in the middle temporal gyrus. The merged overview shows DAPI (blue), ULEX (white), CD8 (red), and CD57 (green). A magnified image shows the separate channels of CD57 and CD8. Scale bar = 1 mm for overview and 10 μm for magnifications. e Bargraph showing the total number of CD8^+^ and CD57^+^ CD8^+^ T cells in the middle temporal gyrus. Bargraphs show mean ± SEM, Mann–Whitney test. **f** Violin plot displaying the concentration of CCL2 in blood plasma using a GLM with age and sex as covariates. **g** Correlation graph showing correlations of CCL2 with sTREM2 and YKL40. Graph shows linear regression ± 95% confidence intervals, Spearman's rho correlation with age/sex correction. **a**-**c** *n* = 35 of control, *n* = 21 of MCI due to AD, *n* = 57 of Dem. **d**-**e** *n* = 5 of control, *n* = 8 AD. **a**-**e**. **f**-**g** *n* = 24 of control, *n* = 19 of MCI due to AD, *n* = 55 of Dem. GLM = multivariate general linear model; Ctrl = Control, MCI = mild cognitive impairment; Dem = dementia; AD = Alzheimer’s disease; TEMRA = effector memory CD8^+^ T cell re-expressing CD45RA

Then, we postulated that the increase in PD1^+^ CD57^+^ CD8^+^ TEMRA cells in patients with MCI-AD might be a component of a recent immune response, which could be reflected by associations between CD8^+^ TEMRA cells and other immune cell subsets in the early stages of the disease. Indeed, Spearman correlation networks that exposed underlying immune connectomes per AD stage showed that significant correlations (*P*_adj_ < 0.05) between CD8^+^ TEMRA cells and other immune cells were most prominent in the MCI stage of AD (Fig. 2a; Additional file 6). Within the complete cohort, higher levels of CD8^+^ TEMRA cells were most strongly correlated to an increase in both CD57^+^ KLRG1^+^ CD4^+^ terminally differentiated effector memory T cells and senescent CD57^+^ gamma-delta (γδ) T cells re-expressing CD45RA (Fig. 2b,c; Additional file 7). Of note, CD8^+^ TEMRA cells correlated negatively to levels of naïve CD4^+^ T cells, regulatory CD4^+^ T cells, and KLRG1^−^ CD57^−^ CD4^+^ central memory-like T cells amongst others (Fig. 2b). We predominantly observed these immune correlations with CD8^+^ TEMRA cells in the MCI-AD and AD dementia groups (Fig. 2c). In contrast, these correlations were considerably weaker and did not attain significance in the control group (Fig. 2c).

CD8^+^ TEMRA cells have been functionally characterized as senescent [32]. Given the challenges in identifying TEMRA cell markers compatible with immunohistochemistry, we opted to probe for the existence of senescent CD8^+^ T cells in AD patient brains. To achieve this, we employed the expression of CD57 as a surrogate marker for T cell senescence. Using a distinct pathological cohort (Additional file 8), we discovered an elevated count of CD8^+^ T and CD57^+^ CD8^+^ T cells in the middle temporal gyrus of AD patients with pathologically confirmed amyloid pathology. Contrarily, minimal CD8^+^ and CD57^+^ CD8^+^ T cells were detected in amyloid-negative or amyloid-low non-demented control patients (Fig. 2d, e). It is important to note that an increase in CD57^−^ CD8^+^ T cells was also observed in the AD brain (Fig. S4a), corroborating previous findings of a general influx of infiltrating CD8^+^ T cells in the AD brain.

A variety of cytokines and chemokines are known to play a role in the recruitment and modulation of immune cells. To investigate mediators that could promote T cell influx in the AD brain, we assessed a broad range of cytokines and chemokines in blood plasma of the cognitively healthy individuals, MCI-AD and AD dementia patients (Additional file 14, Fig. S4b). We observed an increase in CCL2 levels in both MCI-AD and AD dementia patients (Fig. 2f). CCL2 is a key factor in lymphocyte recruitment [33] and is highly produced by Aβ-associated microglia and astrocytes [34, 35]. Indeed, we found that CCL2 positively correlated with CSF markers of neuroinflammation, as chitinase-3-like protein 1 (YKL-40) and soluble triggering receptor expressed on myeloid cells 2 (sTREM-2) in AD dementia (Fig. 2g). A finding that fortifies the link between microglial activation and the recruitment of peripheral immune cells in AD. Moreover, we found a trend in increased perforin levels in MCI-AD patients, and increased CXCL10 levels in AD dementia patients (Fig. S4b). In conclusion, our analysis shows the emergence of intricate immune networks, marked by complex CD8^+^ TEMRA cell immune interactions along with a rise in CD8^+^ T cells expressing markers associated with senescence in AD patient brains. Furthermore, increased peripheral CCL2 levels that are associated with CSF markers of neuroinflammation in AD patients signify the link between neuroinflammation and systemic adaptive immune alterations during the progression of AD.

### Peripheral immune cells correlate with clinical measures for AD pathology and cognitive function

**Fig. 3:**
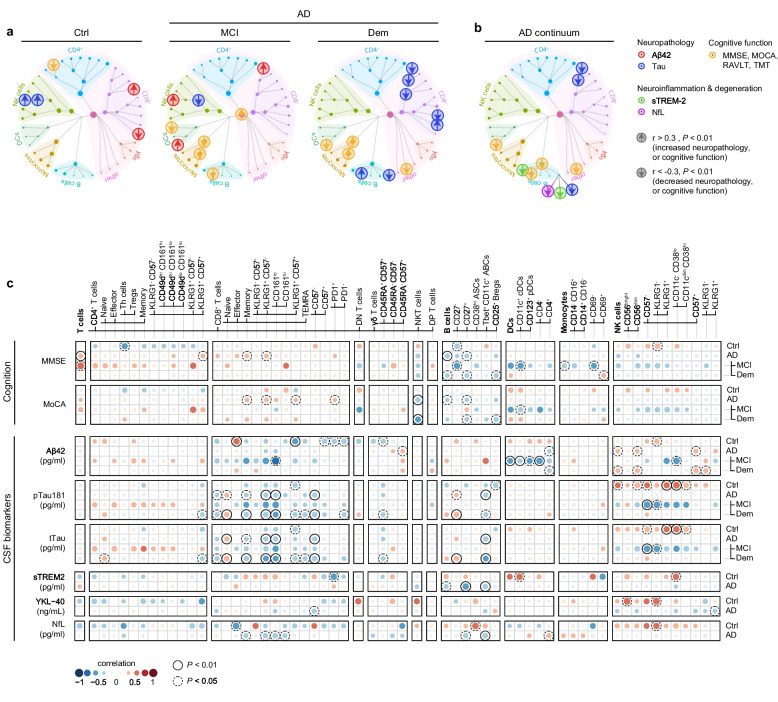
Correlations between peripheral immune subsets and CSF biomarkers for AD pathology and cognition. **a**, **b** Clinical outcome measurements are plotted on top of the immune cell network, where the size of the nodes represents the abundance of the population, and the color of nodes represents the immune parent. Circles corresponds to a relation (*P* < 0.01, Spearman's rho correlation with age/sex correction) between an immune subset and (a) CSF Aβ42 (red), CSF tTau and pTau (blue), cognitive function (yellow) and (b) sTREM-2 (green) and, NfL (purple). Values of CSF Aβ42 and TMT cognitive tests were inverted so that arrows indicate the positive (up) or negative (down) link with CSF biomarkers of AD neuropathology (CSF Aβ42, tTau, and pTau), neuroinflammation or degeneration (CSF sTREM-2 and NfL) and cognitive function (MMSE, MOCA, RAVLT, and TMT).** c** Correlation matrix showing the association between immune cell populations with CSF biomarkers for AD neuropathology or cognition. A partial two-tailed Spearman correlation was performed and controlled for age and sex. *n* = 35 of control, *n* = 21 of MCI due to AD, *n* = 59 of Dem, *n* = 80 of AD. For sTREM2, YKL − 40, Nfl; *n* = 20 of control, *n* = 52 of AD. CSF = cerebrospinal fluid; GLM = multivariate general linear model; Ctrl = Control, MCI = mild cognitive impairment; Dem = dementia; AD = Alzheimer’s disease; Aβ42 = amyloid-beta 1–42; pTau = tau phosphorylated at threonine 181; tTau = total tau; sTREM-2 = soluble triggering receptor expressed on myeloid cells 2; NfL = neurofilament light; MMSE = Mini-Mental State Examination; MOCA = Montreal Cognitive Assessment; RAVLT = Rey Auditory Verbal Learning Tests; TMT = Trail Making Tests

We further investigated age and sex-adjusted associations between immune cell subsets and biomarkers of AD pathology and cognitive function. Subsets of immune cells were correlated to measures for Aβ and tau pathology (CSF-levels of Aβ42, pTau181, and tTau) and cognitive tests including the Mini-Mental State Examination (MMSE) and Montreal Cognitive Assessment (MOCA) for global cognition, Rey Auditory Verbal Learning Tests (RAVLT) for memory and Trail Making Tests (TMT) for attention and executive function (Fig. 3a, S4). In addition, we searched for possible relations between peripheral immune cells and measures for neuroinflammation YKL-40 and sTREM-2, and neurodegeneration (CSF-levels of neurofilament light (NfL)) (Fig. 3a-c). Limited by CSF sample availability, MCI-AD and AD dementia groups were combined in the correlation analysis for YKL-40, sTREM-2, and NfL.

We found many significant relations between immune cell subsets and disease outcome measures (Fig. 3a-c, S4; Additional file 9; Additional file 10). For MCI-AD, we found that most immune associations were established with CSF Aβ42 levels (for example, CD161^lo^ KLRG1^+^ CD57^−^ CD8^+^ effector-like memory T cells ~ CSF Aβ42 levels, *r*_MCI_ = -0.66; Fig. 3a-c). When evaluating dementia due to AD, immune associations with CSF biomarkers for tau pathology were more prominent (for example, CD57^−^ CD8^+^ TEMRA cells ~ CSF pTau levels, *r*_Dem_ = -0.40; Fig. 3a-c). Within the MCI-AD cohort, we identified a negative correlation between both conventional dendritic cells (cDCs) and plasmacytoid dendritic cells (pDCs) with CSF Aβ42 levels (Fig. 3a-c). In AD dementia patients, an intriguing association surfaced, where elevated CSF pTau levels positively correlated with naïve CD8^+^ T cells (*r*_Dem_ = 0.36), while an inverse relationship was observed with various memory CD8^+^ T cell populations and CD57^−^ CD8^+^ TEMRA cells (Fig. 3a-c). Remarkably, natural killer (NK) cells exhibited divergent associations with CSF pTau and tTau pathology when comparing the control group with the MCI-AD cohort (Fig. 3a-c). Specifically, a positive correlation existed between NK cells and tau pathology in the control group, while an inverse correlation was manifested in the MCI-AD group. In addition, we found that immune cell subsets were uniquely, but to a lesser extent, associated with cognitive decline, as measured by different neuropsychological tests, per AD stage (for example, CD14^+^ monocytes ~ RAVLT immediate recall, *r*_MCI_ = -0.64, and *r*_Dem_ = 0.45) (Fig. S5). These results reveal relationships between immunological and disease outcome measures, that are dependent on the AD disease phase. In addition, naïve-like CD27^−^ B cells were related to worse memory scores in MCI-AD (RAVLT delayed recall; *r*_MCI_ = -0.62) and to more CSF pTau and tTau, a biomarker for tauopathy and neurodegeneration, in dementia patients (CSF tTau levels; *r*_Dem_ = 0.36, and CSF pTau levels; *r*_Dem_ = 0.38) (Fig. 3c). While long-lived age-associated effector B cells (ABCs) were associated with less CSF tTau in dementia patients (*r*_Dem_ = -0.40) (Fig. 3c). These findings suggest that the differentiation of naïve B cells into ABCs is associated with less neurodegeneration and cognitive decline. Indeed, ABCs were also negatively associated in AD patients with CSF levels of NfL (*r*_AD_ = -0.38) (Fig. 3b). In addition, we found a negative correlation (*P* < 0.01) between both the abundance of circulating ABCs (*r*_AD_ = -0.37) and CD27^+^ memory-like B cells (*r*_AD_ = -0.47), and levels of sTREM-2 in patients with AD (Fig. 3c). We found no significant correlations with YKL-40. While no causality can be conferred from this data, these findings suggest that the differentiation of naïve B cells into long-lived effector and memory B cells is associated with less neuroinflammation and neurodegeneration. In summary, our results reveal unique associations between various immune cell subsets and indicators of AD pathology and cognitive function, specific to the early and late clinical stages of AD.

### APOE ε4 carriers with AD exhibit a distinct adaptive immune signature

**Fig. 4:**
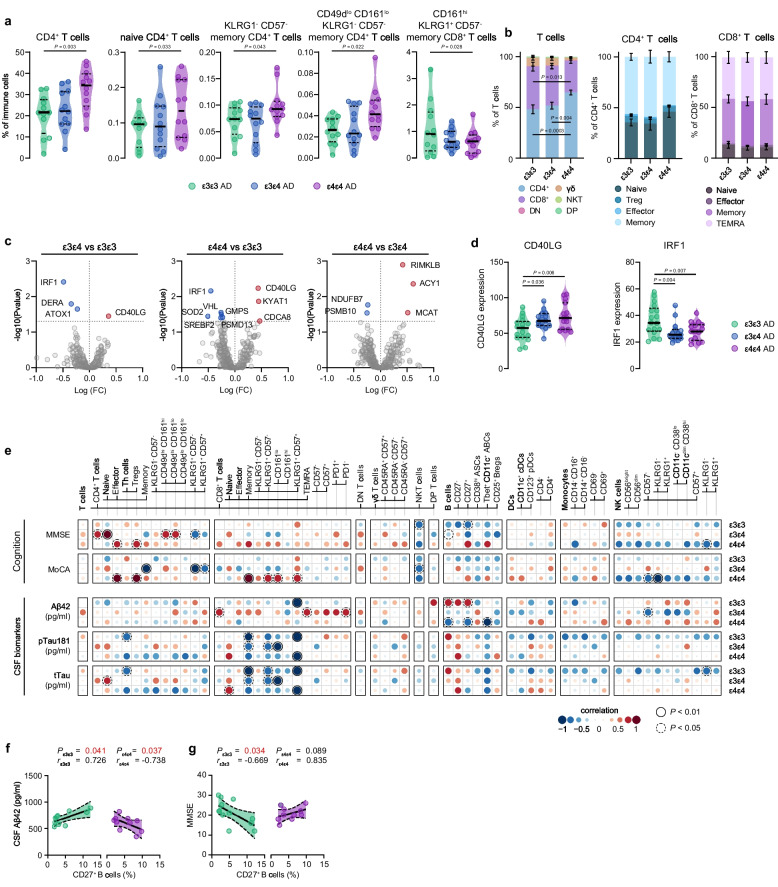
Influence of APOE status on peripheral immunity in AD patients. **a** Violin plots displaying the percentage of the annotated immune cell population out of the total CD45^+^ immune population using a GLM with age and sex as covariates in different APOE genotypes across the AD continuum. **b** Stacked bar graphs showing the percentage of T cell and CD4^+^ T cells subpopulations out of the total T cell and CD4^+^ T cell populations. **c** Volcano plots displaying differentially expressed genes (*P*_unadjusted_ < 0.05) between different APOE genotypes in AD. Red and blue indicate respectively higher and lower expression. **d** Violin plots displaying the expression of the two genes that were differentially expressed in both APOE ε4ε4 and APOE ε3ε4 compared to the APOE ε3ε3 carriers. **e** Correlation matrix showing the association between immune cell populations with CSF biomarkers of AD neuropathology or cognition. A partial two-tailed Spearman correlation was performed and controlled for age and sex. **f**, **g** Correlation graph showing correlations of CD27^+^ B cells with CSF Aβ42 (f) and MMSE (g). **a**, **d** Violin plots showing median ± quartiles. **f**, **g** Graph shows linear regression ± 95% confidence intervals. *n* = 12 ε3ε3 AD, *n* = 12 ε3ε4 AD, *n* = 12 ε4ε4 AD. GLM = multivariate general linear model; AD = Alzheimer’s disease

Recognizing the role of APOE signaling in immune responses, we studied if AD patients with the APOE ε4 allele present a distinctive immune signature. MCI-AD and AD dementia patients were grouped by their APOE genotype into three age-similar categories: ε3ε3, ε3ε4, and ε4ε4 (Additional file 11). Employing a GLM with age and sex as covariates, we discerned that numerous CD4^+^ T cell populations, incorporating both naive and memory subsets, were elevated in the ε4ε4 group compared to the ε3ε3 group (Fig. 4a). Remarkably, the ε3ε4 group often manifested an intermediate effect. Moreover, we noticed a decrease in KLRG1^+^ CD57^−^ CD8^+^ T effector-like memory cells expressing CD161 in the ε4ε4 group relative to the ε3ε3 group (Fig. 4a). While we documented an upsurge in CD4^+^ T cells within T cell clusters, the distribution of CD4^+^ T cell subclusters within CD4^+^ T cells persisted unchanged (Fig. 4b).

As APOE is a crucial contributor to various metabolic pathways utilized by cells to coordinate their function [36], we hypothesized that the immune cells of ε4 carriers could exhibit a distinct metabolic state, potentially influencing the observed variations in immune parameters of ε4 carriers. To scrutinize this hypothesis, we employed the nCounter Metabolic Pathways panel to profile 768 genes participating in cell metabolism and immunology, within PBMCs derived from ε3ε3, ε3ε4, and ε4ε4 AD patients (Additional file 13). Although none of the genes differed significantly post multiple testing correction, we noticed an increase in CD40 ligand (CD40L) in PBMCs sourced from both ε3ε4 and ε4ε4 AD patients compared to ε3ε3 AD patients (Fig. 4c-d). Conversely, interferon (IFN) regulatory factor 1 (IRF1), a strong type I IFN inducer, was diminished in both ε3ε4 and ε4ε4 AD patients relative to ε3ε3 AD patients (Fig. 4c-d). Additionally, our examination disclosed a sequence of other genes influenced by the ε4 allele in peripheral immune cells (Fig. S6a) involved in cell proliferation (CDCA8), energy and lipid metabolism (KYAT1, NDUFB7, ACY1, SREBF2, MCAT), copper transport (ATOX1), proteasome (PSMD13, PSMB10), oxidative stress (SOD1), hypoxia (VHL), and DNA synthesis, repair and post-translational processes (GMPS, DERA, RIMKLB).

We proceeded to explore immune associations with CSF biomarkers for AD pathology as per APOE genotype group (Fig. 4e; Additional file 11). We discovered that CD8^+^ TEMRA cells, and specifically PD1^−^ CD57^+^ CD8^+^ TEMRA cells, correlated positively with CSF Aβ42 levels (implying lower amyloid burden), but only in the ε3ε4 group (Fig. 4e). While CD8^+^ TEMRA cells showed no correlation with alterations in MMSE or MoCA cognitive scores, surprisingly, PD1^−^ CD57^+^ CD8^+^ TEMRA cells were associated with lower scores on the RAVLT tests for episodic memory in ε3ε4 carriers (Fig. S6b). Regardless of the APOE genotype, we discovered that memory CD8^+^ T cells negatively correlated with pTau and tTau CSF levels, reinforcing our earlier findings in the AD dementia group (Fig. 3c). This suggests that higher levels of memory CD8^+^ T cells correlate with reduced Tau pathology. Furthermore, higher levels of memory CD8^+^ T cells were associated with improved scores on the MoCA cognitive test, but only in ε4ε4 carriers (Fig. 4e). In addition, CD27^+^ memory-like B cells were positively correlated with CSF Aβ42 levels, but two copies of the ε4-allele completely reversed this correlation (Fig. 4f). This indicates that elevated levels of CD27^+^ memory-like B cells correlate with lower amyloid pathology in ε3ε3 carriers but higher pathology in the ε4ε4 group. Paradoxically, increased levels of CD27^+^ memory-like B cells also correlated with worsened cognitive function in the ε3ε3 group as assessed with MMSE score (Fig. 4g).

In summary, our findings provide evidence that the ε4 allele significantly modifies the peripheral immune landscape across different clinical stages of AD, influencing a diverse range of metabolic and immunological pathways within peripheral immune cells.

## Discussion

Our study reveals significant variations in the levels of various peripheral immune cells in AD patients over the course of the disease. These results align with findings from prior research (reviewed in [37]) and expand upon them by incorporating aspects of AD progression. Our methodology involved a comprehensive profiling approach, coupled with the assessment of a wide array of clinical biomarkers. In particular, our data shows that higher levels of circulating CD8^+^ T cells, particularly CD57^+^ TEMRA cells expressing PD1, were detected in the MCI stage of AD. Moreover, we found that several peripheral innate and adaptive immune cells are associated with CSF biomarkers of AD pathology and with cognitive decline. Lastly, ε4 carriers with AD showed changes in adaptive immune cell levels and the expression of genes involved in B and T cell activation, providing support for the notion that APOE has a role in adaptive immunity. Altogether, we show alterations in peripheral adaptive immune cells in AD patients that are associated with AD pathology and cognitive function.

CD8^+^ TEMRA cells constitute a preformed effector T cell population with enhanced expression of effector molecules [27, 38], that have superior antiviral capacity [39]. CD57 has been described as a marker for senescence in CD8^+^ TEMRA cells, and CD8^+^ TEMRA cells that express CD57 possess shorter telomeres and decreased proliferative capacity and IFN-γ release compared to their CD57^−^ counterpart [38]. In addition, the CD57^+^ subset shows limited ability to differentiate phenotypically compared to the CD57^−^ subset, while having comparable cytotoxicity [38]. It has been hypothesized that increased levels of CD8^+^ TEMRA cells in AD patients are associated with viral infections [8]. Possibly, non-self-antigens, like viral antigens, initiate and/or exacerbate AD-associated neuroinflammation via molecular mimicry, a process where a foreign antigen shares sequence or structural similarities with self-antigens [40]. Clonally expanded T cells specific to two distinct Epstein-Barr virus (EBV) antigens have been identified in the CSF of one MCI and two AD patients before [8]. Notably, also blood-derived T cells of AD patients were highly clonally expanded and had a reduced TCR diversity [41, 42]. We confirm that levels of CD8^+^ TEMRA cells are increased in patients with AD, and found that these cells start to accumulate in the MCI stage and additionally express PD1 and CD57. PD1 is an inhibitory receptor, that suppresses TCR signaling [28–30] and is quickly upregulated after antigen stimulation [31]. Thus, higher levels of PD1^+^ CD57^+^ CD8^+^ TEMRA cells in the MCI stage of AD suggest recent TCR activation. In addition, the PD1^+^ subset also expressed higher levels of CD161, which is associated with increased effector functions and cytotoxicity in memory CD8^+^ T cells [43]. In a greater pool of MCI patients with varying or normal CSF biomarker levels, effector CD8^+^ T cells were associated with accumulating amyloid pathology, suggesting specificity of this upregulation for MCI-AD. Effector cells are short-lived cells that expand and differentiate during a primary response, and a small subset of these cells eventually differentiates into memory cell precursors to give rise to the pool of long-lived memory T cells [44]. Possibly, a minority of these effector CD8^+^ T cells that increase with amyloid pathology in MCI patients, could be responsible for the rise in CD57^+^ CD8^+^ TEMRA cells observed in patients with MCI and dementia due to AD. In AD patients, these accumulating CD8^+^ TEMRA cells could benefit and protect the individual from viral infections, but might also induce excessive neuroinflammatory responses towards self-antigens as Aβ through molecule mimicry, ultimately accelerating ongoing neurodegenerative processes.

Mapping the immune connectome in AD patients with MCI and dementia can help to elucidate changes in the dynamic balance between immune cell subsets at different stages of AD. We found that CD8^+^ TEMRA cells were positively associated with other terminally differentiated and senescent T cell subsets. In contrast, CD8^+^ TEMRA cells formed immune networks with subsets of CD4^+^ T cells in the MCI stage of AD, while these inter-relations were absent in controls and dementia patients. Notably, levels of regulatory CD4^+^ T cells, which suppress the immune response and maintain homeostasis and self-tolerance, were inversely correlated to levels of CD8^+^ TEMRA cells in AD patients. Regulatory CD4^+^ T cells can control both CD8^+^ T cell activation and differentiation [45, 46]. Early-stage depletion of regulatory CD4^+^ T cells in AD mouse models has been shown to worsen cognitive deficits [47], while enhancing their numbers with treatments like IL-2 can improve cognitive function and reduce Aβ plaque load [48]. In contrast, at later stages in AD mouse models, reducing regulatory CD4^+^ T cell activity leads to decreased plaque deposition and cognitive improvement [49]. Studies also show that Aβ-specific CD4^+^ T cells, especially those with a Th2 phenotype, are beneficial in AD, reducing amyloid levels and improving cognition [50, 51]. Overall, research indicates a complex, stage-dependent role for regulatory and Aβ-specific CD4^+^ T cells in AD pathology. While no direct nor causal relationships can be inferred from these data, we show that a loss of regulatory immune cells is concomitant with increased levels of CD8^+^ TEMRA cells in AD.

Correlations between the abundance of immune cell subsets and CSF biomarkers of AD pathology were mostly dependent on the disease stage. For patients with MCI-AD, immune cell associations with Aβ were most prominent, while immune cell subsets were mostly correlated to tau pathology in the dementia stage of AD. These results support the theory that the buildup of Aβ occurs earlier in the disease progression, which is subsequently followed by tau pathology. They also imply a connection between these two pathologies and the immune system, indicating that changes in the peripheral immune system occur in line with these clinical stages. In patients with AD dementia, we observed an intriguing correlation where elevated CSF pTau and tTau levels positively associated with the abundance of naive CD8^+^ T cells. In contrast, various memory CD8^+^ T cell populations and CD57^−^ CD8^+^ TEMRA cells showed an inverse relationship with CSF Tau and NfL levels, the latter signifying axonal degeneration. This suggests a possible connection between either a decrease in the differentiation of naive CD8^+^ T cells into memory CD8^+^ T cells, or an increase in the conversion of memory CD8^+^ T cells into CD8^+^ CD57^+^ TEMRA cells, and heightened tau pathology and neurodegeneration. These hypotheses are further reinforced by our discovery of a reduction in memory CD8^+^ T cells within the total CD8^+^ T cell fraction in the AD dementia group. Our findings indicate that Tau pathology is the main driver of T cell changes in AD dementia. Previous studies have reported that T cell extravasation is induced by Tau pathology rather than by Aβ pathology and promotes neuroinflammation and cognitive deficits [15, 52]. Furthermore, it was observed that the Tau-associated T cell response is primarily coordinated through dysfunctional microglia [19].

Perhaps surprisingly, we did not observe positive relationships between the abundance of peripheral immune cell subsets and CSF measures of neuroinflammation (sTREM-2 and YKL-40). On the contrary, higher levels of memory-like B cells and long-lived ABCs were associated with decreased neuroinflammation, as indicated by lower CSF levels of sTREM-2. Moreover, ABCs were also negatively related to CSF levels of NfL, and CSF levels of tTau and pTau. ABCs, as defined by CD11c^+^ and Tbet^+^ expression, are a minor population of long-lived effector B cells that accumulate during aging and are associated with viral infections and auto-immunity [53–55]. ABCs display various functional capacities, including secretion of antibodies and activation of T cells, and protection from recurrent viral infection. Previous research into the interplay between B cells and AD pathology has established that the prevalence of memory B cells within the CSF positively correlates with increased Aβ burden in MCI patients, a situation further exacerbated in APOE ε4 allele carriers [56]. Our study mirrors these findings, with high levels of CD27^+^ memory-like B cells being linked to reduced amyloid pathology in ε3ε3 carriers and increased pathology in ε4ε4 carriers. Interestingly, our data also revealed that an elevation in CD27^+^ memory-like B cells correlated with poorer cognitive performance in the ε3ε3 group based on MMSE scoring. This prompts speculation that if CD27^+^ memory-like B cells are involved in amyloid clearance, the beneficial effect of their elevated presence might be overshadowed by the adverse consequences of inflammation mediated by these cells.

While the involvement of APOE in adaptive immunity has been relatively under-explored, our findings provide evidence that the APOE ε4 genotype is linked with alterations in peripheral lymphocytes. Specifically, we found changes in the abundance of CD4^+^ T cell populations, with the ε4ε4 group presenting an increase in both naive and memory subsets compared to ε3ε3 carriers. The correlation between these immune cells and CSF biomarkers of AD pathology, as well as with cognition, varied for certain measures across genotypes, as we previously discussed. At the transcriptomic level, we identified a notable increase (uncorrected *P* < 0.01) in CD40L expression in PBMCs derived from both ε3ε4 and ε4ε4-carrying AD patients, relative to ε3ε3 AD patients. CD40L plays a crucial role in the activation of CD8^+^ T cells through the licensing of DCs by CD4^+^ T cells [57, 58], and is a potent B cell activator [59]. Importantly, IRF1, a strong type I IFNs inducer, was the most downregulated gene in PBMCs derived from both ε3ε4 and ε4ε4 carriers with AD, relative to ε3ε3 AD patients. Together, these findings reveal adaptive immune changes in ε4 carriers, characterized by a decrease in type 1 IFN signaling and an increase in CD40L expression, both of which facilitate T and B cell activation. Furthermore, our investigation unveiled a series of additional genes, influenced by the ε4 allele in peripheral immune cells, that are implicated in energy and lipid metabolism. Others have also shown differences in immune cells derived from ε4 carriers. For example, monocyte-derived dendritic cells from ε4 carriers show increased expression of MHC-II and enhanced accumulation of lipid rafts, both boosting T cell activation [9]. In line with this, an earlier study observed that homozygous APOE ε4 AD subjects harbored the highest number of infiltrated T cells in the hippocampus as compared to other APOE genotypes, although the sample set-up did not allow for proper statistical analysis[15]. A recent study found that APOE, and especially APOE ε4, can induce T cell activation via neuronal MHC-I overexpression [60]. In addition, APOE is found to be involved in the process of lipid antigen presentation by B cells [61]. While limited, these studies show that APOE ε4 can change the adaptive cell response via modulating MHC-I and MHC-II presentation by neurons and antigen-presenting cells respectively, possibly via underlying changes in their immune cell metabolism.

Multiple studies have so far identified an increase in infiltrated adaptive immune cells in the brains of AD patients, and spatial association of these immune cells with neurons, microglia, and Aβ and tau pathology [8, 12–19]. We detected higher numbers of CD8^+^ T cells expressing CD57 in the middle temporal gyrus of AD patients while almost no CD8^+^ T cells were detected in control patients. Cytokines and chemokines are widely recognized for their role in attracting immune cells to the brain and affecting their functionality. In our observations, we noted an increase in peripheral CCL2 in patients with MCI-AD and AD dementia. CCL2 is crucial for lymphocyte recruitment [33], and in particular for memory T cells [62, 63]. We found a positive correlation between CCL2 and CSF levels of YKL-40 and sTREM2, both measures of neuroinflammation, in AD dementia patients. Intriguingly, CCL2 was found to be produced by Aβ-associated microglia and astrocytes [34, 35]. Together, these findings point to a link between neuroinflammation and memory T cell recruitment in AD. Within the brain, CD8^+^ T cells can induce phenotypic changes in neurons and microglia [64], and are involved in axonal degeneration and neuronal killing via MHC-I-dependent interactions [65–68], IFN-γ-mediated inhibition of neural stem cell proliferation [69], synaptic elimination by microglia [70, 71] and associate with a decline in synaptic plasticity and cognition in AD pathology [18, 8]. Lastly, a recent study identified that microglia-mediated infiltration of T cells is a driver of neurodegeneration in tauopathies, but only in the presence of APOE [19]. Interestingly, a recent study uncovered that PD1 expression in brain resident CD8^+^ T cells can direct them towards a regulatory phenotype that restricts mouse AD pathology by inhibiting microglia activation [72]. Furthermore, PD1 expression is essential for the uptake of Aβ, and the abolition of PD1 signaling through a PD1 knockout model exacerbates the development of plaque pathology and cognitive deterioration [73]. We observed that circulating CD57^+^ CD8^+^ TEMRA cells increased expression of the immunosuppressive marker PD1 in MCI-AD, but not in AD dementia. Additionally, lower PD1 expression by CD8^+^ TEMRA cells correlated with increased Aβ and pTau pathology, as indicated by CSF biomarker levels. These observations may imply a transition from regulatory functions to enhanced cytotoxic activity in CD8^+^ TEMRA cells as AD progresses, accompanied by a diminished capacity for Aβ uptake. Collectively, these discoveries illustrate the complex interactions between T cells, microglia, and neuronal cells, potentially triggering a cascade of events that leads from neuronal and network alterations to ultimate cognitive decline.

Over the past decade, numerous therapies aimed at modifying AD progression have been trialed but ultimately failed in clinical settings [74]. Only recently, Aducanumab and Lecanemab emerged as the first FDA-approved disease-modifying drugs for AD. These antibodies slow AD progression by targeting Aβ in its aggregated state (Aducanumab) or as soluble protofibrils (Lecanemab), facilitating the removal of Aβ plaques from the brain [75, 76]. Furthermore, the development of treatments aimed at enhancing the proliferation of systemic regulatory T cells for AD has progressed to clinical trials, demonstrating encouraging preliminary outcomes [77]. The use of adoptive therapy with Aβ-specific regulatory T cells presents another potential therapeutic approach, as it has shown to reduce neuroinflammation and enhance cognitive function in preclinical mouse models of AD [78–80]. Our research identified reduced PD1 expression on CD8^+^ TEMRA cells in AD dementia patients, with lower PD1 expression being associated with increased markers of AD pathology. With previous research underscoring the role of PD1 signaling in attenuating Aβ pathology [72, 73], enhancing PD1 signaling emerges as a promising therapeutic strategy for AD. Nonetheless, any therapeutic attempts must also assess PD1 expression across diverse neuronal populations and its influence on neuronal activities such as excitability, synaptic functionality, and neuroplasticity [81]. Understanding the effects of modifying PD1 signaling on the equilibrium among Aβ clearance, inflammation, and neuronal function is crucial. Additionally, clarifying the distinct molecular mechanisms and functions of PD1 and its ligands in both the immune and neural contexts within AD is imperative, next to searching for effective methods to deliver any PD1 modulating agent to the brain. Finally, multiple sclerosis medications like Natalizumab and Fingolimod are being explored for AD treatment. Natalizumab blocks T cell entry into the brain, thereby reducing microgliosis, Aβ pathology, tau hyperphosphorylation, and enhancing cognition in AD models [82, 83]. Fingolimod, which limits T cell infiltration into the brain, has shown promise in AD models by decreasing pathology and cognitive deficits [84, 85]. Overall, comprehending the role of different T cell subsets in AD is crucial for developing effective immunomodulatory AD treatments.

While we believe that our study contributes significantly to the collective understanding of immunity in AD, it is not without its limitations. We acknowledge the potential shortfall of our 37-marker CyTOF panel in fully capturing all necessary markers to define rare immune subpopulations. Further, our findings imply that changes in immunity are intertwined with early disease processes. However, it is pertinent to note that AD processes can start a decade or more before the onset of visible pathology. Hence, incorporating a pre-clinical AD group could have enhanced our study. In addition, the use of a mixed cell population in our bulk transcriptomic analysis could lead to potential confounding factors due to the variability in the composition of immune cells across different samples. Lastly, our research is primarily descriptive, which inherently limits our ability to derive causative conclusions. Despite these limitations, we maintain that our results substantially enhance the understanding of immunity in AD. While cross-sectional human studies are inherently limited in their capacity to draw definitive conclusions, they serve as crucial preliminary steps, generating hypotheses for more comprehensive and rigorous future investigations.

## Conclusions

We show that CD8^+^ TEMRA cells that express markers of T cell senescence accumulate in AD patients before dementia onset. The prevalence of several innate and adaptive immune cells was associated with biomarkers of AD pathology and with cognitive function, with these associations being influenced by the clinical stage of AD. Finally, we provide evidence that the APOE ε4 genotype is associated with different B and T cell phenotypes. These data emphasize that AD pathophysiology involves dynamic immunological processes that extend beyond the confines of the brain. We believe that future research studies should focus on (1) measuring longitudinal immunological changes over the AD course, (2) investigating if immunological changes can predict disease progression in a preclinical stadium, (3) deciphering which self or non-self-antigens are recognized by the accumulated antigen-experienced CD8^+^ T cells in AD, and (4) elucidating the role of T and B cells in AD pathology. Eventually, an increased understanding of underlying adaptive immune cell mechanisms and their targeted antigens in AD will push therapeutic developments that target the immune system.

### Supplementary Information

**Additional file 1.**

**Additional file 2.**

**Additional file 3.**

**Additional file 4.**

**Additional file 5.**

**Additional file 6.**

**Additional file 7.**

**Additional file 8.**

**Additional file 9.**

**Additional file 10.**

**Additional file 11.**

**Additional file1 2.**

**Additional file 13.**

**Additional file 14.**

**Additional file 15.**

**Additional file 16: Fig. S1.** Cohort characteristics and pre-gating strategy. a. Stacked bargraph showing the number of cells imputed for clustering and cell annotation per donor. b. Pre-gating strategy (1) CyTOF, (2) deselecting cell debris, doublets, and beads, (3) manual removal of unstable flow part 1, (4) usage of the flowCut algorithm, (5) manual removal of unstable flow part 2, (6) debarcoding, (7) live-cell selection, (8) selecting CD45+ immune cells, and (10) batch normalization using CytoNorm. c. Density plot showing the pre-gating of T cells, DCs, monocytes and NK cells, and B cells for further PARC-guided clustering. DC = dentritic cell; NK = natural-killer. Ctrl = Control; MCI = mild cognitive impairment due to Alzheimer’s disease; Dem = dementia due to Alzheimer’s disease; f = female; m = male.

**Additional file 17: Fig. S2.** Heatmap of PARC-guided annotated clusters. Heatmap showing median expression values for each immune cell population. Colors correspond to PARC-guided clustering. Horizontal bars show the percentage of each cluster out of the total number of cells. Heatmap (left) displaying the abundance of each immune cluster in control, MCI, and Dem. Nodes (right) display significant lower (blue) and higher (red) abundance of immune clusters between different experimental groups using a GLM with age and sex as covariates. GLM = multivariate general linear model; *n* = 35 of control, *n* = 21 of MCI due to AD, *n* = 59 of Dem, *n* = 80 of AD; Ctrl = Control, MCI = mild cognitive impairment due to Alzheimer’s disease, Dem = dementia due to Alzheimer’s disease.

**Additional file 18: Fig. S3.** Significant different immune cell subsets separated by sex and correlations with clinical AD parameters. a. Violin plots displaying the percentage of significantly changed immune cell subsets out of the total CD45^+^ immune population using a GLM with age and sex as covariates. b. Correlation matrix showing the association between CD8^+^ T cells immune cell populations with CSF Aβ42, pTau181 and tTau. A partial two-tailed Spearman correlation was performed and controlled for age and sex. *n* = 35 of control, *n* = 21 of MCI due to AD, *n* = 59 of Dem, *n* = 80 of AD. CSF = cerebrospinal fluid; GLM = multivariate general linear model; Ctrl = Control, MCI = mild cognitive impairment; Dem = dementia; AD = Alzheimer’s disease; Aβ42 = amyloid-beta 1-42; pTau = tau phosphorylated at threonine 181; tTau = total tau.

**Additional file 19: Fig. S4.** Immunohistochemical analysis and cytokines and chemokines expression. a. Bargraph showing the total number of CD57^-^ CD8^+^ T cells in the middle temporal gyrus. Bargraphs show mean ± SEM. Mann-Whitney test, *n* = 5 of control, *n* = 8 AD. b. Heatmap (left) showing median expression values of cytokines and chemokines in blood plasma of control, MCI, and Dem. Nodes (right) display significant lower (blue) and higher (red) abundance of cytokines and chemokines between different experimental groups using a GLM with age and sex as covariates. *n* = 24 of control, *n* = 19 of MCI due to AD, *n* = 55 of Dem. GLM = multivariate general linear model; Ctrl = Control, MCI = mild cognitive impairment; Dem = dementia; AD = Alzheimer’s disease.

**Additional file 20: Fig. S5.** Correlations between peripheral immune cells abundance and clinical AD parameters. Correlation matrix showing the association between immune cell populations with CSF biomarkers for cognitive function (RAVLT and TMT). A partial two-tailed Spearman correlation was performed and controlled for age and sex. *n* = 35 of control, *n* = 21 of MCI due to AD, *n* = 59 of Dem, *n* = 80 of AD. Ctrl = Control, MCI = mild cognitive impairment; Dem = dementia; AD = Alzheimer’s disease; RAVLT = Rey Auditory Verbal Learning Tests; TMT = Trail Making Tests.

**Additional file 21: Fig. S6.** Influence of APOE genotype on gene expression and correlations with clinical AD parameters. a. Violin plots display the expression of up and down-regulated genes. b. Correlation matrix showing the association between immune cell populations with CSF biomarkers for cognitive function (RAVLT and TMT). A partial two-tailed Spearman correlation was performed and controlled for age and sex. Volin plots show median ± quartiles; a. *n* = 12 ε3ε3 AD, *n* = 12 ε3ε4 AD, n = 12 ε4ε4 AD. b-c. *n* = 17 ε3ε3 AD, *n* = 15 ε3ε4 AD, *n* = 15 ε4ε4. AD = Alzheimer’s disease; f = female; m = male; RAVLT = Rey Auditory Verbal Learning Tests; TMT = Trail Making Tests.

## Data Availability

The CyTOF dataset generated and analyzed during the current study is available at https://flowrepository.org, ID: FR-FCM-Z6LK, name: CYTOF_AD_PBMCs_Olst. Additional information necessary for the reanalysis of the data reported in this manuscript is available from the corresponding author upon request.

## Abbreviations

AD: Alzheimer’s disease
MCI: Mild cognitive impairment
ABCs: Age-associated effector B cells
APOE: Apolipoprotein
Aβ: Amyloid-beta
Aβ42: Amyloid-beta 1–42
cDCs: Conventional dendritic cells
CSF: Cerebrospinal fluid
CSB: Cell staining buffer
CyTOF: Cytometry by time-of-flight
DCs: Dendritic cells
GLM: Multivariate general linear model
IFN: Interferon
IRF1: Regulatory factor 1
MMSE: Mini-Mental State Examination
MOCA: Montreal Cognitive Assessment
MRI: Magnetic resonance imaging
NfL: Neurofilament light
NIA-AA: National Institute on Aging-Alzheimer's Association
NK: Natural killer
PARC: Phenotyping by Accelerated Refined Community
PBMCs: Peripheral blood mononuclear cells
PD1: Programmed cell death protein 1
pDCs: Plasmacytoid dendritic cells
pTau: Phosphorylated tau
RAVLT: Rey Auditory Verbal Learning Test
sTREM-2: Triggering receptor expressed on myeloid cells 2
TCR: T cell receptor
TEMRA: T effector memory cells re-expressing CD45RA
TMT: Trail Making Tests
tTau: Total tau
YKL-40: Chitinase-3-like protein 1
γδ: Gamma-delta
