# Supplementary figures and images for: Adaptive immune changes associate with clinical progression of Alzheimer’s disease
Source: Mol Neurodegener. 2024 Apr 24;19:38. doi: 10.1186/s13024-024-00726-8 (PMC11044380; doi:10.1186/s13024-024-00726-8)

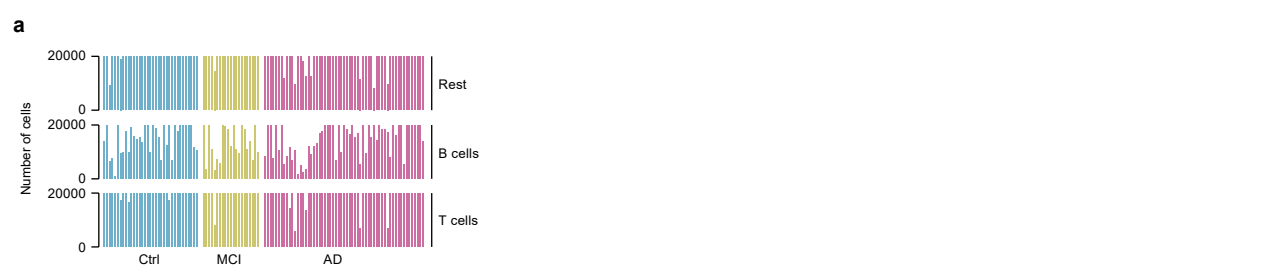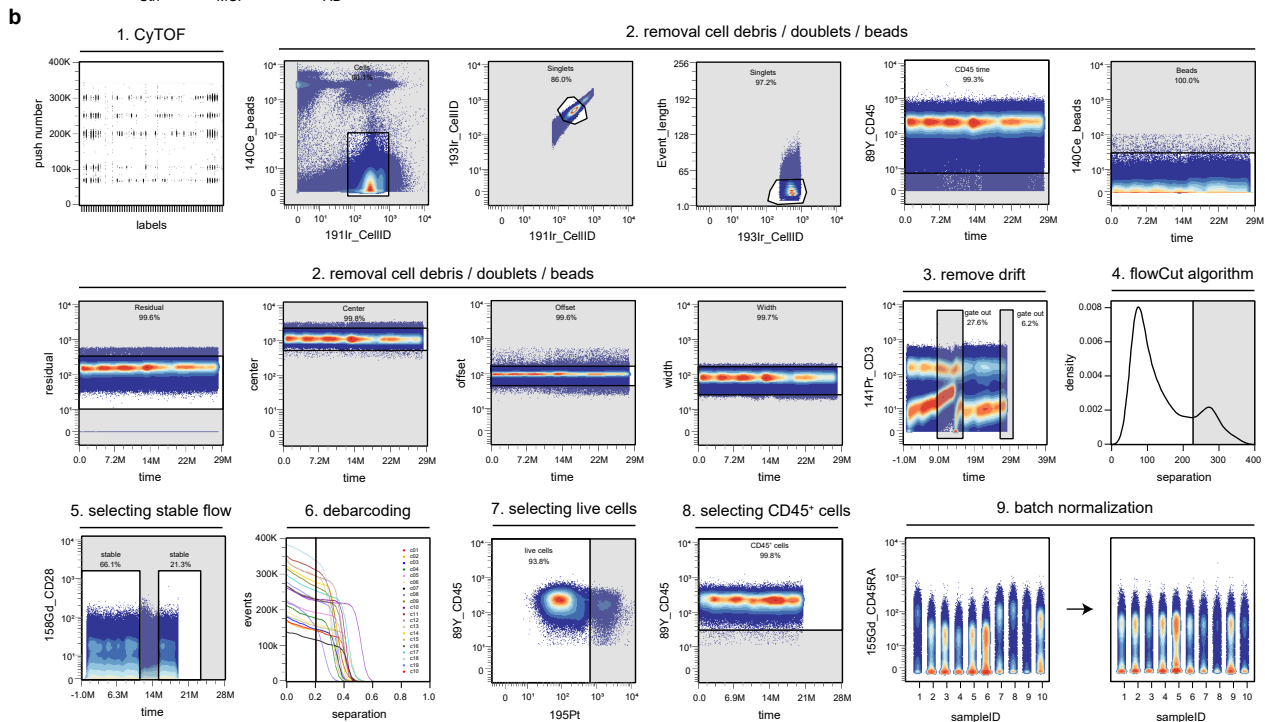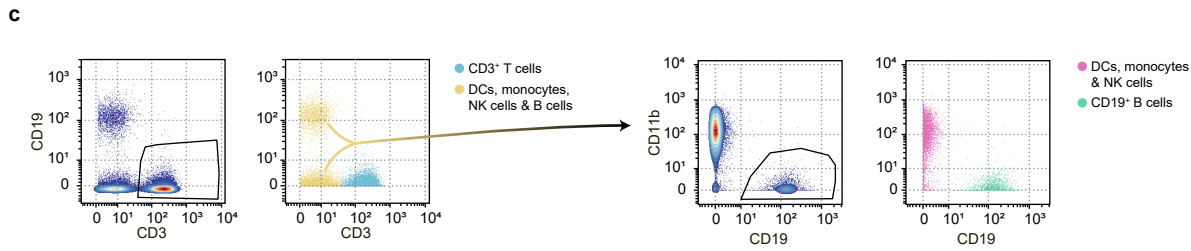

Supplement: Supplementary file 16 — Additional file 16: Fig. S1. Cohort characteristics and pre-gating strategy. a. Stacked bargraph showing the number of cells imputed for clustering and cell annotation per donor. b. Pre-gating strategy (1) CyTOF, (2) deselecting cell debris, doublets, and beads, (3) manual removal of unstable flow part 1, (4) usage of the flowCut algorithm, (5) manual removal of unstable flow part 2, (6) debarcoding, (7) live-cell selection, (8) selecting CD45+ immune cells, and (10) batch normalization using CytoNorm. c. Density plot showing the pre-gating of T cells, DCs, monocytes and NK cells, and B cells for further PARC-guided clustering. DC = dentritic cell; NK = natural-killer. Ctrl = Control; MCI = mild cognitive impairment due to Alzheimer’s disease; Dem = dementia due to Alzheimer’s disease; f = female; m = male. [file 13024_2024_726_MOESM16_ESM.pdf]

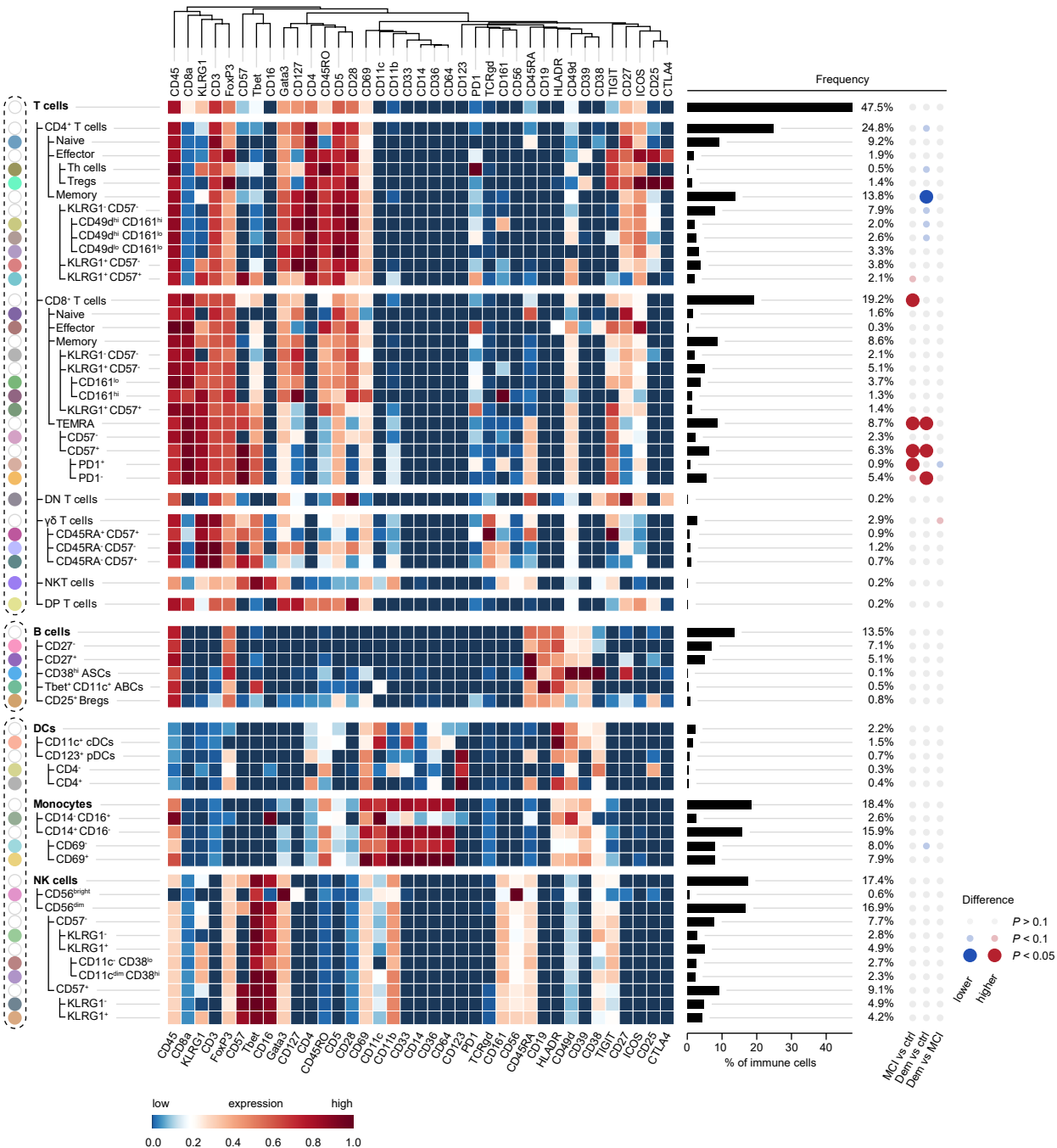

Supplement: Supplementary file 17 — Additional file 17: Fig. S2. Heatmap of PARC-guided annotated clusters. Heatmap showing median expression values for each immune cell population. Colors correspond to PARC-guided clustering. Horizontal bars show the percentage of each cluster out of the total number of cells. Heatmap (left) displaying the abundance of each immune cluster in control, MCI, and Dem. Nodes (right) display significant lower (blue) and higher (red) abundance of immune clusters between different experimental groups using a GLM with age and sex as covariates. GLM = multivariate general linear model; n = 35 of control, n = 21 of MCI due to AD, n = 59 of Dem, n = 80 of AD; Ctrl = Control, MCI = mild cognitive impairment due to Alzheimer’s disease, Dem = dementia due to Alzheimer’s disease. [file 13024_2024_726_MOESM17_ESM.pdf]

**a**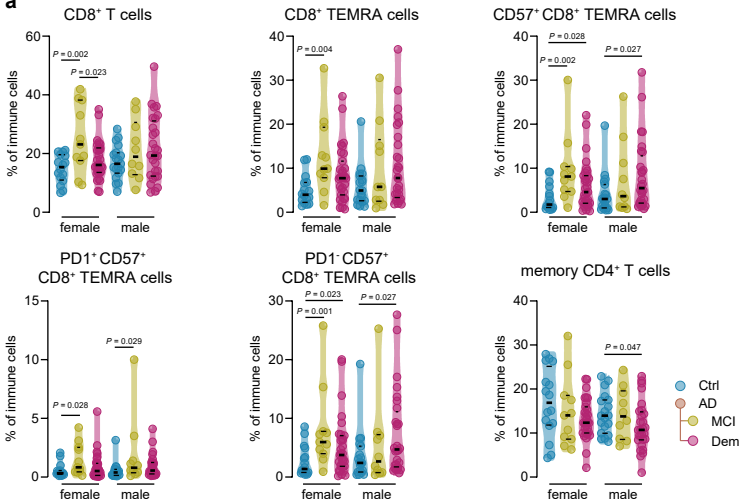**b**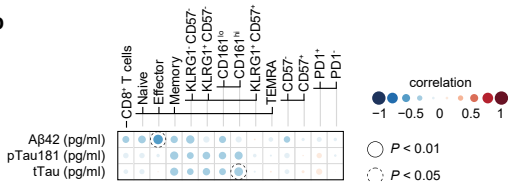

Supplement: Supplementary file 18 — Additional file 18: Fig. S3. Significant different immune cell subsets separated by sex and correlations with clinical AD parameters. a. Violin plots displaying the percentage of significantly changed immune cell subsets out of the total CD45+ immune population using a GLM with age and sex as covariates. b. Correlation matrix showing the association between CD8+ T cells immune cell populations with CSF Aβ42, pTau181 and tTau. A partial two-tailed Spearman correlation was performed and controlled for age and sex. n = 35 of control, n = 21 of MCI due to AD, n = 59 of Dem, n = 80 of AD. CSF = cerebrospinal fluid; GLM = multivariate general linear model; Ctrl = Control, MCI = mild cognitive impairment; Dem = dementia; AD = Alzheimer’s disease; Aβ42 = amyloid-beta 1-42; pTau = tau phosphorylated at threonine 181; tTau = total tau. [file 13024_2024_726_MOESM18_ESM.pdf]

**a**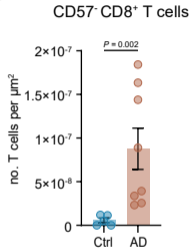**b**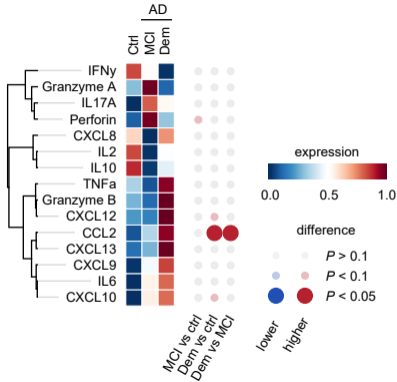

Supplement: Supplementary file 19 — Additional file 19: Fig. S4. Immunohistochemical analysis and cytokines and chemokines expression. a. Bargraph showing the total number of CD57- CD8+ T cells in the middle temporal gyrus. Bargraphs show mean ± SEM. Mann-Whitney test, n = 5 of control, n = 8 AD. b. Heatmap (left) showing median expression values of cytokines and chemokines in blood plasma of control, MCI, and Dem. Nodes (right) display significant lower (blue) and higher (red) abundance of cytokines and chemokines between different experimental groups using a GLM with age and sex as covariates. n = 24 of control, n = 19 of MCI due to AD, n = 55 of Dem. GLM = multivariate general linear model; Ctrl = Control, MCI = mild cognitive impairment; Dem = dementia; AD = Alzheimer’s disease. [file 13024_2024_726_MOESM19_ESM.pdf]

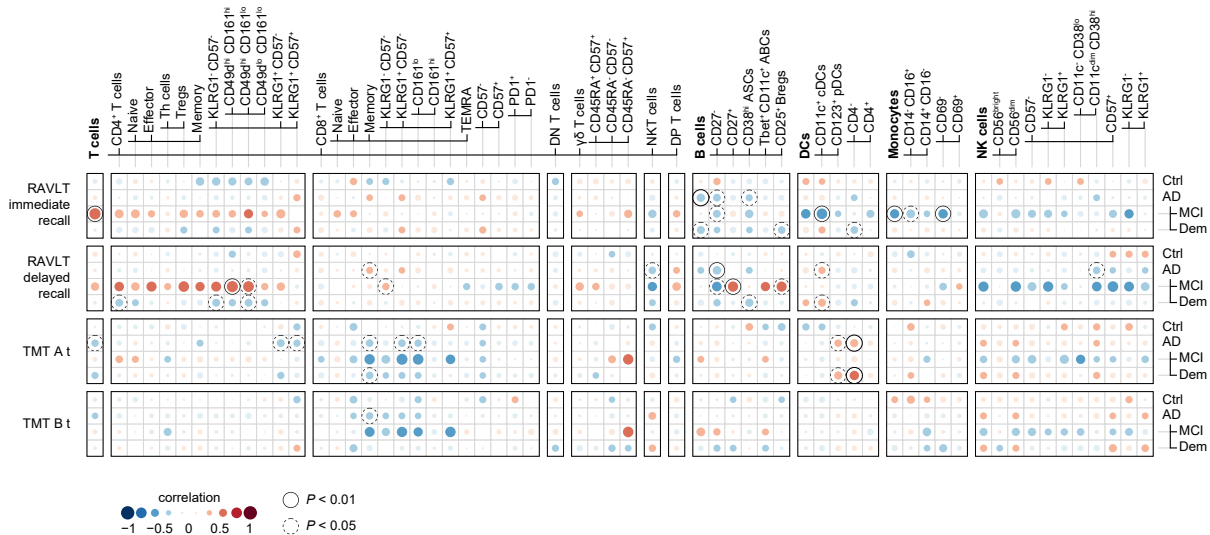

Supplement: Supplementary file 20 — Additional file 20: Fig. S5. Correlations between peripheral immune cells abundance and clinical AD parameters. Correlation matrix showing the association between immune cell populations with CSF biomarkers for cognitive function (RAVLT and TMT). A partial two-tailed Spearman correlation was performed and controlled for age and sex. n = 35 of control, n = 21 of MCI due to AD, n = 59 of Dem, n = 80 of AD. Ctrl = Control, MCI = mild cognitive impairment; Dem = dementia; AD = Alzheimer’s disease; RAVLT = Rey Auditory Verbal Learning Tests; TMT = Trail Making Tests. [file 13024_2024_726_MOESM20_ESM.pdf]

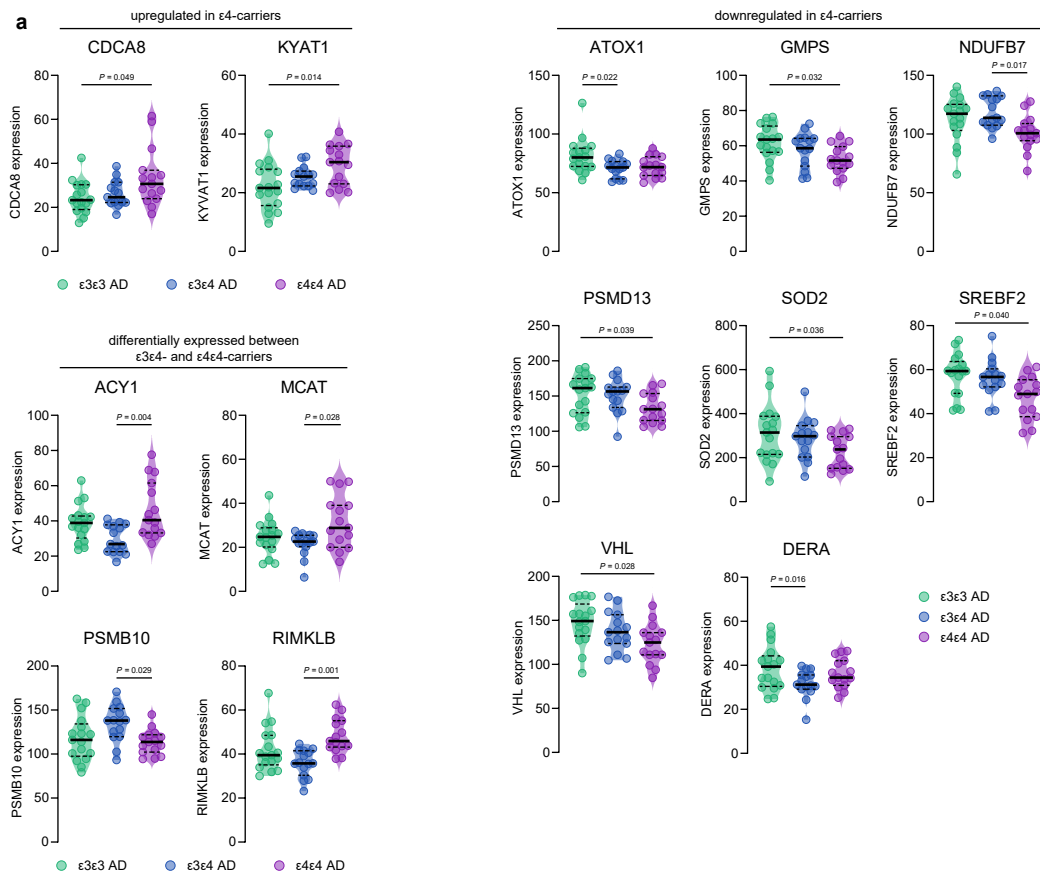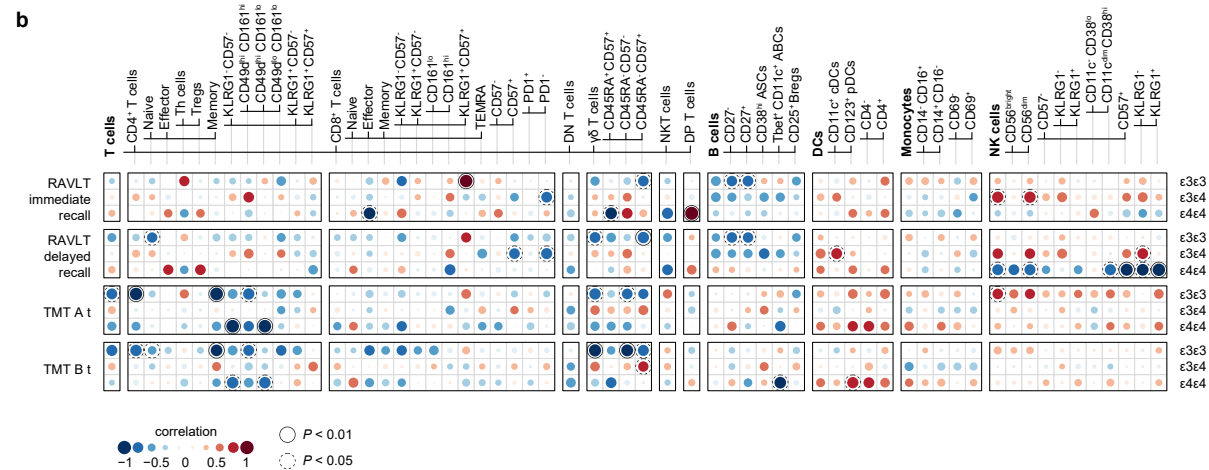

Supplement: Supplementary file 21 — Additional file 21: Fig. S6. Influence of APOE genotype on gene expression and correlations with clinical AD parameters. a. Violin plots display the expression of up and down-regulated genes. b. Correlation matrix showing the association between immune cell populations with CSF biomarkers for cognitive function (RAVLT and TMT). A partial two-tailed Spearman correlation was performed and controlled for age and sex. Volin plots show median ± quartiles; a. n = 12 ε3ε3 AD, n = 12 ε3ε4 AD, n = 12 ε4ε4 AD. b-c. n = 17 ε3ε3 AD, n = 15 ε3ε4 AD, n = 15 ε4ε4. AD = Alzheimer’s disease; f = female; m = male; RAVLT = Rey Auditory Verbal Learning Tests; TMT = Trail Making Tests. [file 13024_2024_726_MOESM21_ESM.pdf]
